# A Study on Staging Cystic Echinococcosis Using Machine Learning Methods

**DOI:** 10.3390/bioengineering12020181

**Published:** 2025-02-13

**Authors:** Tuvshinsaikhan Tegshee, Temuulen Dorjsuren, Sungju Lee, Dolgorsuren Batjargal

**Affiliations:** 1 Department of Information Technology, School of Information and Communication Technology, Mongolian University of Science and Technology, Ulaanbaatar 13341, Mongolia; tegsheetuvshinsaikhan@gmail.com; 2 Department of Biology, School of Bio-Medicine, Mongolian National University of Medical Sciences, P.O. Box 48/111, S. Zorig Street-3, Ulaanbaatar 14210, Mongolia; temuulen@mnums.edu.mn; 3 Department of Software, Sangmyung University, Cheonan-si 31066, Republic of Korea

**Keywords:** image processing, deep learning, classification, disease diagnosis

## Abstract

Cystic echinococcosis (CE) is a chronic parasitic disease characterized by slow progression and non-specific clinical symptoms, often leading to delayed diagnosis and treatment. Early and precise diagnosis is crucial for effective treatment, particularly considering the five stages of CE as outlined by the World Health Organization (WHO). This study explores the development of an advanced system that leverages artificial intelligence (AI) and machine learning (ML) techniques to classify CE cysts into stages using various imaging modalities, including computed tomography (CT), ultrasound (US), and magnetic resonance imaging (MRI). A total of ten ML algorithms were evaluated across these datasets, using performance metrics such as accuracy, precision, recall (sensitivity), specificity, and F1 score. These metrics offer diverse criteria for assessing model performance. To address this, we propose a normalization and scoring technique that consolidates all metrics into a final score, allowing for the identification of the best model that meets the desired criteria for CE cyst classification. The experimental results demonstrate that hybrid models, such as CNN+ResNet and Inception+ResNet, consistently outperformed other models across all three datasets. Specifically, CNN+ResNet, selected as the best model, achieved 97.55% accuracy on CT images, 93.99% accuracy on US images, and 100% accuracy on MRI images. This research underscores the potential of hybrid and pre-trained models in advancing medical image classification, providing a promising approach to improving the differential diagnosis of CE disease.

## 1. Introduction

It has been well established that echinococcosis is a zoonotic infectious disease belonging to the cestodes family, caused by parasitizing tapeworms that parasitize and form cysts in organs such as the liver and lungs. This pathogen is composed of five species, among which Echinococcus granulosus and Echinococcus multilocularis are the most common, each causing a different form of echinococcosis, which is usually termed CE or alveolar echinococcosis (AE). Currently, Echinococcosis is being recognized as one of the most significant and re-emerging zoonotic diseases that are being overlooked. Yang et al. [1] estimated that CE cases worldwide could reach 236,628 by 2030, which represents an increase of 13.63 percent over 2019. In the last few decades, Echinococcus granulosus has become one of the most significant threats to the public health of nations throughout the world. Several mammals are affected by this parasitic disease, and the infection is transmitted to humans by the ingestion of food and water that have been contaminated with feces from infected dogs and other carnivores. Hydatid cysts, caused by this unicellular parasite, typically remain asymptomatic for extended periods, silently growing within the human body. These cysts, as they enlarge, apply pressure to surrounding tissues and organs, significantly reducing the quality of life of the patient as a result of the impact they have on them. In the event that these cysts rupture, they can cause serious complications to a person’s health. The diagnosis of hydatid cysts often involves medical imaging techniques such as X-rays, CT, US, and MRI. Currently, these cysts are classified manually by Mongolian doctors in order to ensure their accurate location. An accurate and timely classification of these cysts is crucial for ensuring appropriate treatment and reducing the risk of complications associated with them. In 2023, we proposed a hybrid method [2] for classifying CE into three categories—active, inactive, and transition. Initially, we implemented a system in C#.NET that utilizes the ML.Net Model Builder [3] using deep learning neural network (DNN) algorithms to perform image-based classifications. In our previous study, in comparison to manual classification methods, the application of ML and AI has demonstrated better accuracy in automated classification compared with the use of manual methods. To further enhance accuracy, we aimed to implement and utilize many different methods based on the Convolutional Neural Network (CNN) [4] architecture, such as Inception-V3 [5], MobileNet-v2 [6], VGG-16 [7], VGG-19 [8], and Artificial Neural Network (ANN) architecture, such as Feedforward Neural Networks (FNN) [9], and also ViT [10] models for classification, detection, and segmentation of cysts using Python [11]. We aim to classify various clinical imaging modalities, including US, CT, and MRI, using a variety of deep learning (DL) models. Considering the unique characteristics of different DL architectures (such as CNNs, RNNs, and Transformers), we customized specific parameters for each model across three distinct datasets.

## 2. Literature Review

ML is increasingly applied to medical diagnostics, particularly in imaging. It has the potential to automate, accelerate, and improve diagnostic accuracy by recognizing patterns that may not be easily discernible to the human eye. Upon conducting a thorough review of numerous scholarly articles, we were quite astonished to ascertain that certain methodologies we employed were similarly utilized within these investigations, which was noteworthy to acknowledge. Presented below is a summary of select research endeavors we analyzed. These studies highlight the significance of interdisciplinary approaches and their impact on advancing our understanding of complex phenomena.

Although specific ML applications in CE are still emerging, the use of ML in parasitic disease diagnostics could be highlighted. Recent works that focus on ML and parasitic diseases should be discussed, such as Kumar et al. [12], who focused on automating parasite detection using DL. Lee et al. [13] was to evaluate the detection and diagnosis of three types of odontogenic cystic lesions (OCLs)—odontogenic keratocysts, dentigerous cysts, and periapical cysts—using dental panoramic radiography and cone beam computed tomographic (CBCT) images based on a deep CNN. In the case of hydatid cysts, Yildrim et al. [14] utilized CNN and K-nearest neighbors (KNN) methods for classifying CT images. However, upon comparing the results, it appears that our outcomes are superior to those presented in this work. Also, Sulyok et al. [15] uses DL, specifically using CNN, VGG-19, and ResNet-18, similar to our approach. Nevertheless, their results are also inferior to ours in terms of quality. In addition to the fact that they employed ResNet-18 in their study, our work utilized ResNet-50, which may have accounted for the improved performance in our study over theirs. For US image processing, Wu et al. [16] applied Inception, ResNet-18, and VGG-19 models. Our experiments show higher accuracy than theirs. In terms of prediction accuracy, their models with pre-trained weights (fine-tuning) ranged from 88.2 to 90.6%. The best accuracy was obtained by the VGG-19.

According to the literature review, we listed common ML techniques used in disease staging. For example, CNNs, Support Vector Machines (SVMs), and Random Forests are often applied in medical image classification. Relevant literature is listed below. Litjens et al. [17] did a review of DL in medical imaging, with a focus on how CNNs are applied to classify diseases in radiological images. Esteva et al. [18] showed the effectiveness of DL in dermatological disease classification, illustrating the broad applicability of ML techniques. ML in Other Infectious Diseases: While studies specific to CE may be limited, you can draw from research in similar infectious diseases or liver diseases where ML was used for diagnosis. Some examples include Mendel et al. exploring ML approaches for liver disease diagnosis using radiological imaging. Kermany et al. [19] demonstrated how ML can be used to diagnose infections using ocular scans, providing insight into how similar methods could apply to CE.

## 3. Problem Statement

Our study specifically addresses the differential diagnosis of CE, aiming to distinguish between the three stages of CE cyst—active, transitional, and inactive—each of which has distinct clinical and radiological characteristics. Also, the task of selecting the best DL model for the classification is inherently complex, especially when dealing with datasets from heterogeneous imaging modalities such as CT, US, and MRI.

We proposed to address this challenge by evaluating *M* DL models, each trained with different *H* hyper-parameter configurations. These models are working on small-sized, modality-specific *D* datasets, where each dataset presents its own set of challenges in terms of variability, noise, and feature representations inherent to the imaging modality.

Given the small sample sizes typical of medical imaging datasets, the models must be capable of learning discriminative features despite limited data availability. The performance of each model is evaluated using *r* multi-dimensional evaluation metrics, including accuracy, loss, precision, recall, AUC–ROC, sensitivity, specificity, F1 score, and AUC–PR (Area Under the Precision-Recall Curve), which are critical for assessing model efficacy in real-world clinical decision-making. These metrics not only provide insight into the model’s ability to generalize across unseen data but also offer a nuanced understanding of its performance with respect to the often imbalanced nature of medical datasets. Specifically, metrics such as sensitivity and specificity are of paramount importance in the medical domain, as they directly influence the model’s ability to detect both the presence and absence of disease, which is crucial for accurate diagnosis. Moreover, the challenge is further exacerbated by the variability between imaging modalities, which can affect feature extraction, representation learning, and ultimately, the model’s diagnostic accuracy. A systematic and rigorous approach is needed to evaluate the trade-offs in performance across modalities, hyperparameters, and evaluation metrics, ensuring that the selected model provides the best possible clinical outcomes while balancing the underlying complexities of the data.

## 4. The Proposed Solution

### 4.1. Classification of Hydatid Cyst

A serological test or US examination can be used to determine the initial diagnosis of CE in patients. It is possible, however, that small cysts cannot be detected through US scanning due to their size. According to Gharbi and WHO-IWGE, in order to obtain a more detailed analysis of the imaging results, specialists interpret the results using the classification criteria that have been developed. In 1981, Gharbi et al. [20] developed a classification system to classify hepatic cysts based on their morphological characteristics of the cysts observed on US imaging, such as the content, wall appearance, presence of daughter cysts, and calcification (see Table 1).

This system was later refined by the WHO in 2001, resulting in the WHO-IWGE standardized classification that is given below (see Table 2). As per the recommendations of the WHO [21], cysts of unknown origin can be divided into three primary types, which can be further subdivided into five stages according to the progression of the cyst. CE cysts can be classified into three types: “active”, “inactive”, and “transitional”. The CE1 and CE2 cysts are categorized as “active” cysts, while the CE3 cysts are regarded as “transitional”. In contrast, the CE4 and CE5 cysts are categorized as “inactive”.

**Figure 1:**
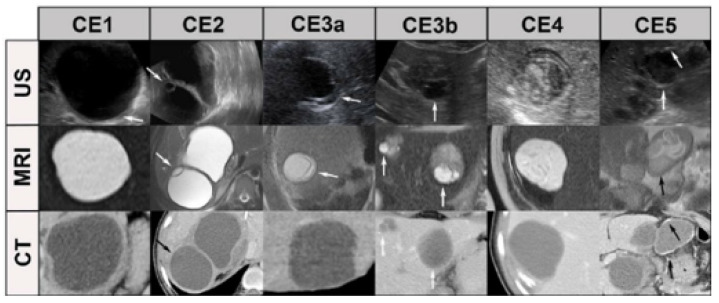
Imaging examples and categories.

A cyst that is classified as transitional (CE3) can be further divided into two subcategories: CE3a [22] (with detached endocysts) and CE3b (containing clear vesicles). Some studies have found that CE3a cysts tend to be inactive, whereas CE3b cysts are active. US imaging can also be employed to monitor the progression of the disease. For patients who have undergone treatment, follow-up examinations should be conducted every 3–6 months and then annually thereafter. Our objective was to classify CE based on US imaging using the latest classification system developed by WHO. We found that US imaging is an effective tool for classifying CE cysts. In addition, US imaging can be used to monitor the disease’s progression and the effectiveness of the treatment. Figure 1 shows how the classification aligns across different imaging modalities such as US, CT, and MRI. Each modality complements the others, with US being the primary staging tool, CT providing detailed structural evaluation, and MRI offering additional soft tissue detail.

### 4.2. Classification Based on Imaging

In some cases, a definitive diagnosis cannot be established using US alone. It is therefore possible that MRI and CT may be necessary for the diagnosis. This is especially relevant for patients who are obese, who have cysts located below the diaphragm, who have secondary infections of the cysts, who have complications in the form of biliary fistulas, and who have cysts that have spread beyond the abdominal cavity. Imaging techniques such as CT and MRI are especially valuable for preoperative and postoperative examinations. MRI is preferred over CT for diagnostic and follow-up purposes due to its superior effectiveness.

US, MRI, and CT scanning are all used to visualize pathological conditions within the human body that need to be treated. It should be noted, however, that MRI is more effective in assessing the condition of soft tissues, while CT is more suitable for assessing the condition of bones and other dense structures, such as muscles and joints. CT scans are a form of radiography that utilize X-rays to penetrate soft tissues and reflect off dense structures, creating high-resolution, highly detailed images of the body. These images must be interpreted by specialized physicians to determine the stage and progression of CE. In Table 3, we summarized imaging modalities. However, due to neglect of this disease, there are few specialists with the necessary expertise in our country. Therefore, as discussed in the following section, it is crucial to develop intelligent systems that can accurately determine the stages of CE cysts and monitor the disease using high-resolution MRI and CT images. As a result of these advanced technologies, it can become much easier to diagnose CE and manage it, compensating for the shortage of specialists in medicine.

### 4.3. ML Methodologies

Hydatid disease (a parasitic infection caused by Echinococcus) can be diagnosed and classified using ML. We selected a diverse set of models (CNNs, VGG16, VGG19, ResNet50, etc.) to leverage their unique strengths as shown in Table 4. Furthermore, models were chosen based on their proven performance in the literature, suitability for our dataset characteristics, and task requirements. Additionally, we conducted empirical evaluations to validate our selections. In order to perform classification tasks, these methods use medical imaging, clinical data, or molecular data.

We used ML approaches to classify hydatid disease based on CT, MRI, and US image data. One of the challenges in classifying hydatid disease is the variability in image quality and resolution, which can affect the accuracy of the model. Additionally, distinguishing between hydatid disease and other similar cystic lesions in imaging can be difficult, leading to potential mis-classification. Furthermore, the limited availability of labeled data for training can hinder the development of robust classification algorithms. Table 4 presents an overview of all the selected ML methods, their structures, and applications, as well as their respective advantages and disadvantages.

The challenges in medical image analysis are complex and varied:

1. **Data diversity:** Ensuring the availability of diverse medical image datasets without compromising patient privacy and confidentiality poses a significant challenge. The high sensitivity of personal health information necessitates careful handling and secure storage practices.
2. **High variability:** Medical images exhibit vast variability due to differences in patient demographics, imaging modalities, and pathological conditions. This variability necessitates robust algorithms capable of generalizing across diverse datasets and conditions.
3. **Interpretability:** The complexity of medical images often makes interpretation challenging. Clinicians need clear, actionable insights from automated analysis systems, emphasizing the importance of developing models capable of providing transparent and understandable explanations for their decisions.
4. **Performance Metrics:** Evaluating the performance of medical image analysis models requires metrics that align with clinical utility and relevance. In addition to technical accuracy, metrics should also encompass clinical results and the delivery of healthcare services.

Addressing these challenges involves leveraging advancements in ML, such as DL models tailored for medical imaging, while adhering to stringent ethical and regulatory standards regarding data privacy and interpretability in healthcare settings.

## 5. Experimental Evaluation

### 5.1. Study Area and the Experimental Fields

Geographically positioned between the Russian Federation and the People’s Republic of China, Mongolia has an approximate population of 3.5 million inhabitants. It is estimated by Bold et al. [23] that the prevalence could be roughly sixteen cases for every one hundred thousand persons in our country. Also, the country’s extensive landscapes and nomadic lifestyle contribute to distinctive epidemiological patterns of CE, thereby making it a significant public health concern. Approximately 30% of the Mongolian population engages in nomadic or semi-nomadic lifestyles, with a substantial reliance on animal husbandry practices.

In the past decade, there has been a marked increase in the number of patients suspected of having CE in Mongolia. Nevertheless, the omission of CE from the national surveillance framework results [24] in inadequate official statistical data. Numerous cases of CE have been confirmed through surgical intervention, frequently affecting younger individuals. The majority of infections are located in the hepatic region (88%), while fewer cases involve the pulmonary system (11%) and the central nervous system (1%).

Multiple socio-economic factors contribute to the rising incidence of CE, including the expansion of livestock farming and a lack of public health education concerning the transmission and prevention of this parasitic disease. These factors underscore the urgent necessity for targeted health initiatives and policies to alleviate the risk and impact of CE in Mongolia [25].

### 5.2. Sampling and Experimental Design

From April 2016 to March 2018, a field-based cross-sectional study was conducted to examine the prevalence and risk factors of CE in Mongolia. In five Mongolian provinces (Bayan-Ulgii, Umnugovi, Khuvsgul, Sukhbaatar, and Khovd), including 39 soums, 1993 people underwent US examination. According to WHO-IWGE expert recommendations, all CE positive cases were classified. There were 187 abdominal cyst positive cases out of 1993 participants. The 187 cases were categorized as 98 CL, 4 AE, and 85 CE. In the majority of CE cases (85), there were US positive hepatic cases; 34.1% (29) were active (CE1, CE2), 29.4% (25) were transitional (CE3a, CE3b), and 36.5% (31) were inactive. There were 13 cases reported in US cyst-negative patients who had previously had cysts removed due to CE diagnosis. Using chest CT, two cases were diagnosed with lung CE.

The outcomes of Temuulen et al. [26] highlight the necessity of adopting a multifaceted approach when managing CE. It suggests that the combination of imaging and serological data can potentially lead to improved patient outcomes. This improvement can come from the early detection of the disease and the application of personalized treatment strategies that are tailored to each patient’s specific condition. In addition to this, the research brings to light the critical need for continuous education and training for healthcare providers. This training should focus on understanding the complexities involved in serological testing and the correct interpretation of imaging results. By enhancing their knowledge and skills related to these essential diagnostic tools, healthcare professionals can significantly improve the accuracy of their diagnoses. This improvement is vital for the successful implementation of effective treatment interventions, which are necessary for ultimately enhancing patient care and outcomes in cases of CE.

We aimed to conduct a comprehensive study on the application of AI in the medical field, particularly in utilizing advanced imaging techniques such as MRI, CT scans, and high-resolution US. The focus was on correlating clinical findings with radiological evidence of CE. This research holds significant potential for enhancing the understanding and diagnosis of echinococcosis, a condition that can lead to severe complications if left untreated. For this study, we meticulously collected data from various imaging facilities and resources, published papers that provided authentic images of CE in patients confirmed to have the disease. To facilitate the analysis and testing of the imaging data, we utilized Python [11], a robust programming language renowned for its extensive libraries and tools tailored to data science and ML applications. Custom scripts were developed to automate the image processing pipeline, enabling efficient management of large volumes of data. This automation ensured a systematic approach to data analysis, allowing us to rigorously assess correlations between clinical manifestations and imaging characteristics with precision and consistency.

Moreover, every model we constructed and every test procedure we executed during the analysis was meticulously documented in accordance with project requirements. This thorough documentation process encompassed detailing the methodologies employed, the parameters selected for analysis, and the outcomes of each step. Such comprehensive documentation is essential for ensuring the reproducibility of our results and providing transparency in our research methodology, thereby reinforcing the reliability and integrity of our findings. By integrating sophisticated imaging techniques with advanced automated processing and analysis, our study aims to offer valuable insights into the diagnosis and management of CE. The correlation of clinical findings with radiological evidence, through this comprehensive approach, has the potential to enhance patient outcomes and contribute to the development of improved treatment protocols for this challenging parasitic disease.

### 5.3. Data Collection

**$D_{1}$—CT dataset:** we utilized a comprehensive dataset comprising 2416 CT images classified into CE1-CE2 (active stage) with 792 images, CE3 (transitional stage) with 444 images, and CE4-CE5 (inactive stage) with 1180 images, sourced from the Kaggle [27] internet repository.

**$D_{2}$—US dataset:** This dataset was sourced from Professor Temuulen’s previous study [26] (Supplementary Material 1). The test data includes 85 original US images of CE-positive cases, with the following distribution: 34.1% (29 images) representing the active stage (CE1, CE2), 29.4% (25 images) representing the transitional stage (CE3a, CE3b), and 36.5% (31 images) representing the inactive stage.

**$D_{3}$—MRI dataset:** Due to the unavailability of MRI images from Mongolia, we conducted a comprehensive literature review and identified nine papers [28,29,30,31,32,33,34,35,36] that included verified and pre-labeled images (Supplementary Material 2). The demo dataset consists of MRI images from CE-positive cases, with 14 images available for each class. Detailed information about the literature review and the selected images is provided in Table 5.

An overview of all the collected experimental datasets for each modality (CT, MRI, and US) is summarized in Table 6.

### 5.4. Data Pre-Processing

In our CT dataset, we had three classes: 792 images for the active stage, 444 images for the transitional stage, and 1180 images for the inactive stage, as summarized in Table 7. The dataset was divided into training and testing subsets using an 80:20 split, ensuring that 80% (1933 images) of the data was allocated for training, while the remaining 20% (483 images) was reserved as the original test set. To address the class imbalance in the training set, we applied SMOTE combined with stratified K-fold cross-validation. The minority classes (active and transitional) were oversampled within each fold until they matched the number of images in the majority class (inactive). This resulted in an augmented training set with 944 images per class, totaling 2832 images in each training fold, while the validation and test sets remained unaltered. This approach ensured the robustness of the evaluation process, maintained the integrity of the test set, and significantly improved model performance by mitigating class imbalance and leveraging stratified sampling for better generalization.

In the case of US and MRI modalities, the prepared dataset is insufficient for robust training and testing purposes. To address this limitation, data augmentation is essential to expand and enhance the dataset. For medical images, particularly those depicting cysts or other structural abnormalities, it is imperative to use data augmentation techniques that preserve the morphological and diagnostic features of the images. These techniques ensure that the augmented data accurately represents the original distribution while increasing the diversity of the dataset to improve model performance and generalization.

**Figure 2:**
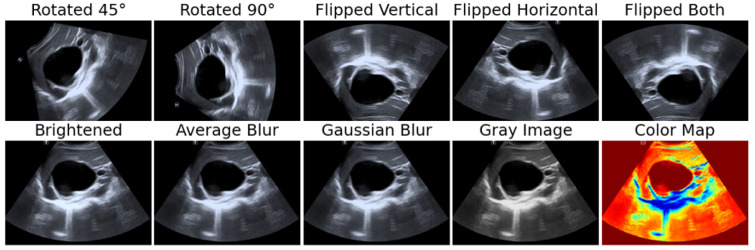
Example of data augmentation applied to the US dataset, demonstrating the use of 10 different augmentation techniques to enhance data diversity.

We enhanced the diversity of the US dataset and improved the robustness of our model by applying ten data augmentation techniques using the OpenCV [37] library. These techniques included rotations (45°,90°), flips (vertical, horizontal, and both), blurring (simple average blur and Gaussian blur), color space transformations (gray_img() and color_img()), and brightness-contrast adjustments (bright_img()). Figure 2 demonstrates the application of these augmentation methods on a selected CE1 cyst image, highlighting the variety introduced into the dataset to strengthen the model’s performance and reduce overfitting. These augmentations simulate real-world variations that the model might encounter, helping to mitigate susceptibility to adversarial attacks. Each augmentation modifies the image by altering its visual characteristics or adjusting parameters such as brightness, contrast, or color representation. To maintain the integrity of the original images, we carefully set parameters like alpha (contrast factor), beta (brightness factor), and kernel size (for filtering) to minimal values where appropriate.

- By adjusting the alpha parameter between 0.8 and 1.2 (alpha=random.uniform(0.8,1.2)), we maintain a balance where the contrast is changed enough to introduce variability but not so much that the image quality is significantly degraded.
- Using beta=0 is useful when we want to vary the contrast of the image but keep the brightness constant. This is especially important in tasks like data augmentation, where we may want to simulate different lighting conditions without changing the overall brightness of the image.
- By using a (3, 3) or (5, 5) kernel, we add a blur effect that subtly alters the image, making it more varied for training purposes while minimizing the loss of critical features. This helps ensure the image quality is preserved, and the model is still exposed to meaningful variations in the data.

Following data augmentation, the dataset, consisting of 935 images, was split into training and testing subsets using an 80/20 ratio, as shown in Table 8. To address class imbalance during evaluation, we employed stratified K-fold sampling. This technique ensures that each fold in the cross-validation process contains the same proportion of each class as the original dataset, providing a balanced representation of classes during both training and validation. By utilizing stratified K-fold, the model is trained on multiple subsets of the data, which enhances its ability to generalize and improves performance, particularly when dealing with imbalanced datasets.

To augment MRI images, we specifically applied rotation. It is crucial to avoid excessive angles (e.g., beyond 90 degrees) as they may introduce unrealistic distortions. Rotations within the range of ±45 degrees are typically safe and effective, allowing for variability while preserving the structural integrity of the images. To generate exactly 100 augmented images, the step size and rotation range were determined using the following formula:

(1) Step_Size=(Number_of_Images−1)/Range_of_Rotation

where the following is true:

- Range_of_Rotation=90 degrees (from −45 to +45 degrees)
- Number_of_Images=100
- The −1 in the formula ensures that the range is divided into 99 intervals, resulting in exactly 100 images (including the original).

**Figure 3:**
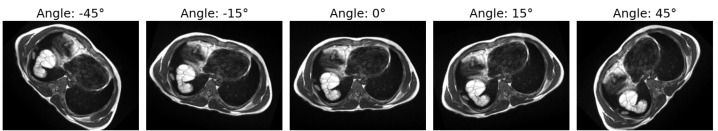
Example of data augmentation applied to the MRI dataset, demonstrating the use of various angle rotation techniques to enhance data diversity.

We rotated the image in increments of approximately 0.91 degrees from −45 to +45 degrees to generate 100 images as shown in Figure 3.

Rotating MRI images as part of a well-balanced data augmentation strategy can enhance the robustness of the model. However, it is crucial to ensure that such rotations do not distort important clinical features or alter the anatomical structure. The augmented data are utilized for training, while the original images are kept aside for testing, as shown in Table 9. This approach is typically effective in improving model robustness and generalization.

We decided against augmenting data using Generative Adversarial Networks (GANs) due to potential challenges associated with GAN-generated images. Such images may introduce artifacts or unrealistic features that do not accurately mirror real medical conditions, especially in complex domains like medical imaging. This can result in misleading outcomes, reduced generalization, and ultimately hinder model performance. Moreover, given the small size of our dataset, training a GAN on a limited or non-representative dataset may produce images that do not properly reflect the true distribution of real-world data, leading to a shift that could adversely affect the model’s performance during testing. Additionally, in the medical field, the use of synthetic data, particularly GAN-generated images, is subject to regulatory oversight, as clinical use of artificial data requires thorough validation to ensure it does not negatively impact patient outcomes.

### 5.5. Performance Metrics

In this study, we utilized a total of nine evaluation metrics to assess the performance of the models. The first six metrics—accuracy, loss, precision, F1 score, recall, and AUC–ROC—are commonly used in general model evaluation. These metrics provide a comprehensive view of the model’s overall performance across different datasets. The remaining three metrics—sensitivity, specificity, and weighted F1 score—hold particular significance in the medical domain. These metrics are essential for evaluating the model’s ability to accurately distinguish between relevant classes, especially in scenarios involving imbalanced data or medical diagnostics, where accurately identifying different stages of disease is crucial. In the following sections, we will provide a detailed explanation of each metric. The formulas and explanations presented below describe the evaluation metrics used to assess the performance of the 10 classification models on each dataset. These metrics serve as the foundation for evaluating the models in this study:

- ${\mathrm{TP}}_{j,i,k}$: true positives for model $M_{j}$ on dataset $D_{i}$ in fold *k*—instances that are correctly predicted as positive.
- ${\mathrm{TN}}_{j,i,k}$: true negatives for model $M_{j}$ on dataset $D_{i}$ in fold *k*—instances that are correctly predicted as negative.
- ${\mathrm{FP}}_{j,i,k}$: false positives for model $M_{j}$ on dataset $D_{i}$ in fold *K*—instances that are incorrectly predicted as positive.
- ${\mathrm{FN}}_{j,i,k}$: false negatives for model $M_{j}$ on dataset $D_{i}$ in fold *k*—instances that are correctly predicted as negative.

**1. Accuracy:** $A_{j,i,k}$ represents the accuracy of model $M_{j}$ on the dataset $D_{i}$ for fold *k*:

(2) $$A_{j,i,k}=\frac{{\mathrm{TP}}_{j,i,k}+{\mathrm{TN}}_{j,i,k}}{{\mathrm{TP}}_{j,i,k}+{\mathrm{TN}}_{j,i,k}+{\mathrm{FP}}_{j,i,k}+{\mathrm{FN}}_{j,i,k}}$$

**2. Loss:** $L_{j,i,k}$, represents the categorical cross-entropy loss based on multi-class classification problems.

(3) $$L_{j,i,k}=-\frac{1}{n_{j,i,k}}\sum_{s=1}^{n_{j,i,k}}\sum_{c=1}^{C}y_{s,c}\log\left({\hat{y}}_{s,c}\right)$$

where the following is true:

- $N_{j,i,k}$: total number of samples in fold *k* for dataset $D_{i}$.
- *C*: total number of classes.
- $y_{s,c}$: binary indicator (0 or 1) whether sample *s* belongs to class *c*.
- ${\hat{y}}_{s,c}$: predicted probability of sample *s* belonging to class *c* by model $M_{j}$.

**3. Precision** $P_{j,i,k}$ measures the ability of the $M_{j}$ model to avoid false positives in *k* fold on the $D_{i}$ dataset, i.e., how many of the predicted positive instances are actually correct made by the model $M_{j}$.

(4) $$P_{j,i,k}=\frac{TP_{j,i,k}}{TP_{j,i,k}+FP_{j,i,k}}$$

**4. Recall**: $R_{j,i,k}$ measures the ability of the model $M_{j,i,k}$ to capture all relevant instances, particularly for the minority class. It shows how many of the actual positive instances were correctly identified.

(5) $$R_{j,i,k}=\frac{TP_{j,i,k}}{TP_{j,i,k}+FN_{j,i,k}}$$

**5. F1 score**: $F_{j,i,k}$ is the harmonic mean of precision $P_{j,i,k}$ and recall $R_{j,i,k}$, providing a single metric to evaluate the model when there is class imbalance. It balances both precision and recall by considering their combined effect.

(6) $$F1_{j,i,k}=2\cdot \frac{P_{j,i,k}\cdot R_{j,i,k}}{P_{j,i,k}+R_{j,i,k}}$$

**6. AUC–ROC**: The Area Under the Receiver Operating Characteristic Curve (AUC–ROC) evaluates the model’s ability to distinguish between classes across different thresholds. It is calculated as the area under the ROC curve, which plots the True Positive Rate (Recall) against the False Positive Rate (FPR).

(7) $${\mathrm{AUC}}_{j,i,k}=\int_{0}^{1}\mathrm{TPR}\left(t\right)\;d\mathrm{FPR}\left(t\right)$$

where the following is true:

- TPR(t): True Positive Rate (also known as sensitivity) at threshold *t*, calculated as
  $$\mathrm{TPR}=\frac{\mathrm{TP}}{\mathrm{TP}+\mathrm{FN}}$$
- FPR(t): False Positive Rate at threshold *t*, calculated as
  $$\mathrm{FPR}=\frac{\mathrm{FP}}{\mathrm{FP}+\mathrm{TN}}$$
- *t*: classification threshold.

The AUC–ROC score ranges from 0 to 1, where the following is true:

- AUC–ROC=1: perfect classification.
- AUC–ROC=0.5: random guess (no discrimination capability).

Specificity and sensitivity (recall) are also highly important in clinical applications. A model with high specificity (true negatives) and high sensitivity (true positives) is preferred.

**7. Sensitivity:** $S_{j,i,k}$ and recall $R_{j,i,k}$ have the same Formula (5), as they both measure the proportion of actual positive instances that are correctly identified by the model. A higher sensitivity means fewer false negatives, which is crucial in clinical diagnostics.

**8. Specificity:** $Sp_{j,i,k}$, also known as the true negative rate (TNR), measures the proportion of actual negatives that are correctly identified by the model. It is important for minimizing false positives, which can lead to unnecessary treatments or tests. Specificity is calculated as

(8) $$S_{j,i,k}=\frac{{\mathrm{TP}}_{j,i,k}}{{\mathrm{TP}}_{j,i,k}+{\mathrm{FN}}_{j,i,k}}$$

Since the F1 score combines precision and recall (sensitivity), the weighted F1 score will reflect both the model’s performance on the common and rare classes, providing a more comprehensive metric for clinical evaluation.

**9. Weighted F1 score** $WF_{j,i,k}$ is an important metric for clinical image classification tasks, especially when handling class imbalances or multi-class problems. In medical diagnostics, such as classifying cystic disease into stages like active, transitional, and inactive, a model must be able to correctly identify all stages. The weighted F1 score takes both the precision and recall of each class into account while considering the relative importance or prevalence of each class.

(9) $$WF_{j,i,k}=\frac{\sum_{k=1}^{C}N_{p}\cdot F_{c,j,i,k}}{\sum_{c=1}^{C}N_{c}}$$

where $F_{c,j,i,k}$ is the F1 score for class *c* for model $M_{j}$ on dataset $D_{i}$ in fold *k*, and it is calculated as

(10) $$F_{c,j,i,k}=2\cdot \frac{P_{c,j,i,k}\cdot R_{c,j,i,k}}{P_{c,j,i,k}+R_{c,j,i,k}}$$

where the following is true:

- $F_{c,j,i,p}$ is the F1 score for class *c* for model $M_{j}$ on dataset $D_{i}$ in fold *k*.
- $P_{c,j,i,k}$ is the precision for class *c* for model $M_{j}$ on dataset $D_{i}$ in fold *k*.
- $R_{c,j,i,k}$ is the recall (or sensitivity) for class *k* for model $M_{j}$ on dataset $D_{i}$ in fold *k*.
- $N_{c}$ is the number of samples in class *c*.

We utilize stratified five-fold cross-validation to ensure a fair and balanced evaluation of models across multiple datasets, while SMOTE addresses class imbalance by oversampling the minority classes during training. By calculating a variety of performance metrics, the algorithm offers a comprehensive assessment of each model’s effectiveness, enabling more reliable comparisons and improvements.

At the end of *K*-fold cross-validation, we compute the average accuracy and average loss over all folds to get a more reliable measure of model training performance. For example, for a five-fold cross-validation with 30 epochs, you can calculate the average accuracy across all five folds after training for 30 epochs. The formula for the average accuracy across all folds is

(11) $$Ave.Accuracy=\frac{1}{k}\sum_{i=1}^{k}Accuracy_{i}$$

where *k* is the number of folds (in our case, k=5), $Accuracy_{i}$ is the accuracy of the model in the *i*th fold after completing 30 epochs.

(12) $$Avg.Loss=\frac{1}{k}\sum_{i=1}^{k}Loss_{i}$$

where *k* is the number of folds.

After each fold *k*, you need to calculate the average of each metric (such as accuracy, precision, recall, F1 score, sensitivity, specificity, and AUC–ROC) across all five folds. This can be done after processing all folds for a given model $M_{j}$ on a given dataset $D_{i}$.

To calculate the average of each metric over the *k* folds for model $M_{j}$ on dataset $D_{i}$, we write the general equation as follows:

(13) $$Ave.\mu_{j,i}=\frac{1}{k}\sum_{ii=1}^{k}\mu_{j,i,k}$$

where the following is true:

- $\mu_{j,i,k}$ represents the value of a specific metric (e.g., accuracy, loss, etc.) for model $M_{j}$, dataset $D_{i}$, and fold *k*.
- The summation is performed over all five folds to compute the average.

Normalize each metric (accuracy, precision, recall, mean F1 score, AUC–ROC, sensitivity, specificity, weighted F1 score) across all models for each dataset ($D_{i}$) using the standard min–max normalization Equation (14):

(14) $${Norm.\mu }_{j,i}=\frac{{Avg.\mu }_{j,i}-\min\left(Avg.\mu \right)}{\max(Avg.\mu )-\min(Avg.\mu )}$$

where Avg.μ refers to the average value of a particular metric (such as accuracy, sensitivity, specificity, F1 score, AUC–ROC) across all models $M_{j}$ for a specific dataset $D_{i}$. Specifically, it is the average of that metric over all the models evaluated on that dataset.

For the loss metric, the normalization is inverted, as lower loss is better, as shown in Equation (15).

(15) $${Norm.L}_{j,i}=1-\frac{L_{j,i}-\min\left(L\right)}{\max(L)-\min(L)}$$

Equations (14) and (15) standardize all your metrics to a scale between 0 and 1, where higher values are better for most metrics, and lower values are better for loss.

Calculate the weighted average for each model $M_{j}$ on dataset $D_{i}$ based on the following metrics and their weights:

(16) $$\begin{array}{c} {Score}_{j,i}=w_{A}\cdot {Norm.A}_{j,i}+w_{L}\cdot {Norm.L}_{j,i}+w_{P}\cdot {Norm.P}_{j,i}+w_{R}\cdot {Norm.R}_{j,i}+\\ w_{F}\cdot {Norm.F}_{j,i}+w_{AUC}\cdot {Norm.AUC}_{j,i}+w_{S}\cdot {Norm.S}_{j,i}+w_{Sp}\cdot {Norm.Sp}_{j,i}+\\ w_{WF}\cdot {Norm.WF}_{j,i}=\sum_{u=1}^{9}w_{u}\cdot {Norm.\mu }_{u,j,i} \end{array}$$

where the following is true:

- $w_{u}$ is the weight for the *u*-th metric.
- $Norm.\mu_{u,j,i}$ is the normalized value of the *k*-th metric for model $M_{j}$ on dataset $D_{i}$.

To assign weights $w_{u}$ to the nine metrics, with three metrics having high impact (higher weights) and six metrics having lower impact (lower weights), we consider clinical context. High-impact metrics (with higher weights) should be the metrics that are most critical for our case, metrics like sensitivity, specificity, and weighted F1 score, because they directly affect the reliability and performance of a model in diagnosing medical conditions.

Low-impact metrics (with lower weights) might not be as critical as the high-impact ones. These could be accuracy, precision, mean F1 score, loss, etc., which are useful for general model evaluation. In our case we assigned $w_{A}=0.05$, $w_{L}=0.1$, $w_{P}=0.05$, $w_{R}=0.05$, $w_{F}=0.05$, $w_{AUC}=0.1$, $w_{S}=0.2$, $w_{Sp}=0.2$, and $w_{WF}=0.2$ for each metric, and their sum is 1.

To calculate the overall score for each model $M_{j}$ by averaging the scores across all datasets $D_{1},D_{2},$ and $D_{3}$, we can use the following equation:

(17) $${FinalScore}_{j}=\frac{1}{3}\sum_{i=1}^{3}{Score}_{j,i}$$

where $Score_{j,i}$ is the weighted score of model $M_{j}$ on dataset $D_{i}$. The summation is performed across all three datasets, and the result is averaged by dividing by 3.

To select the best model $M^{*}$ based on the highest overall score, we can use the following equation:  

(18) $$M^{*}=\arg\max_{j}\left({FinalScore}_{j}\right)$$

where the following is true:

- $M^{*}$ is the best model.
- $M_{j}$ represents each model.
- The overall score for each model is calculated as previously shown (averaging across all datasets $D_{1},D_{2},$ and $D_{3}$.
- argmax selects the model $M_{j}$ that has the highest overall score.

The algorithm for selecting the best model is presented below (see Algorithm 1). It involves training multiple models on each dataset, evaluating their performance using the defined metrics, normalizing the results, and then computing the overall score for each model to identify the best performer.

This algorithm begins by processing three distinct datasets ($D_{1}$, $D_{2}$, $D_{3}$), each containing imbalanced samples from three distinct classes: active, transitional, and inactive. The size of each dataset may vary, denoted as $n_{1}$, $n_{2}$, and $n_{3}$, respectively. A total of ten machine learning models, denoted as $M_{1},M_{2},\cdots ,M_{10}$, are evaluated. Each model is configured with its respective hyper-parameters (drop-out rate, learning rate, epoch, batch size, regularization, and optimizer), denoted as $H_{1},H_{2},\cdots ,H_{6}$.

To address class imbalance, the Synthetic Minority Over-sampling Technique (SMOTE) is applied to the training dataset, generating synthetic samples for the minority classes, thus enhancing the model’s ability to learn from underrepresented classes. The algorithm first computes the class distribution for each dataset, then applies stratified five-fold cross-validation to maintain the proportional distribution of each class across all five folds. This ensures that the representation of the classes in both the training and validation sets mirrors the distribution in the original dataset, which is particularly important in the context of imbalanced datasets. During the training phase, SMOTE is utilized to oversample the minority classes (transitional and active) in the training set, thereby balancing the class distribution and reducing the model’s potential bias towards the majority class (inactive).

Lines 8 to 36 detail the Model Training and Evaluation phase. In this phase, each model is trained on the training set of each fold, while the validation set is used to evaluate its performance. Evaluation metrics such as accuracy, precision, recall, F1 score, confusion matrix, and AUC–ROC are computed for each fold. Upon completing evaluations across all five folds, the algorithm calculates the average performance metrics for each model. This cross-validation approach provides a robust and generalized estimate of model performance, minimizing the risk of overfitting to any specific fold or subset of data. The final output includes the average values of these metrics for each model across all datasets, offering a comprehensive assessment of performance with a focus on addressing class imbalance effectively.

In the algorithm, Lines 37 to 40 focus on model selection. After calculating the average performance metrics for all models, the best model is identified based on a predefined evaluation metric. The model with the highest average score for the selected metric is chosen as the optimal model for the dataset.
**Algorithm 1** Best model for CE cyst classification across three datasets with normalized metrics and weighted evaluation1:**Input:**2:   Datasets $D_{1},D_{2},D_{3}$ with $n_{1},n_{2},n_{3}$ samples, each sample belonging to one of three classes (active, transitional, and inactive)3:   Models $M_{1},M_{2},\cdots ,M_{10}$, each with hyperparameters $H_{1},H_{2},\cdots ,H_{6}$.4:   SMOTE for addressing class imbalance during training.5:   Performance metrics: accuracy (*A*), loss (*L*), precision (*P*), recall (*R*), sensitivity (*S*), specificity (Sp), F1 score (*F*), and AUC–ROC(AUC).6:**Output:**7:   Best model $M^{*}$, based on performance metrics and weighted scores across all datasets.8:**Model Evaluation:**9:**for** each model $M_{j}$ for j=1,2,⋯,10 **do**10:    **for** each dataset $D_{i}$ for i=1,2,3 with $n_{i}$ samples **do**11:        Set hyperparameters for model $M_{j}$ as $H_{j}$12:        Calculate the class distribution (active, transitional, and inactive) for dataset $D_{i}$13:        Apply stratified five-fold to preserve the class distribution across the five folds14:        **for** each fold *k* from 1 to 5 **do**15:           Let $D_{\mathrm{train}}$ be the union of the four folds, excluding fold *k*16:           Let $D_{\mathrm{val}}$ be fold *k* (validation set)17:           Apply SMOTE to oversample the minority classes in $D_{\mathrm{train}}$ to balance18:           Train model $M_{j}$ on $D_{\mathrm{train}}$ using hyperparameters $H_{j}$19:           Evaluate model $M_{j}$ on $D_{\mathrm{val}}$20:           Calculate accuracy $A_{j,i,k}$ for fold *k* on dataset $D_{i}$ using Equation (2)21:           Calculate loss $L_{j,i,k}$ for fold *k* on dataset $D_{i}$ using Equation (3)22:           Calculate precision $P_{j,i,k}$ for fold *k* on dataset $D_{i}$ using Equation (4)23:           Calculate recall $R_{j,i,k}$ for fold *k* on dataset $D_{i}$ using Equation (5)24:           Calculate mean F1 score $F_{j,i,k}$ for fold *k* on dataset $D_{i}$ using Equation (6)25:           Calculate AUC–ROC $AUC_{j,i,k}$ for fold *k* on dataset $D_{i}$ using Equation (7)26:           **for** each class c=1,2,3 **do**27:               Calculate sensitivity for class *c*: $S_{c,j,i,k}=\frac{{\mathrm{TP}}_{c}}{{\mathrm{TP}}_{c}+{\mathrm{FN}}_{c}}$28:               Calculate specificity for class *c*: ${\mathrm{Sp}}_{c,j,i,k}=\frac{{\mathrm{TN}}_{c}}{{\mathrm{TN}}_{c}+{\mathrm{FP}}_{c}}$29:               Calculate F1 score for class *c*: ${F1}_{c,j,i,k}=\frac{2\cdot {\mathrm{Precision}}_{c,j,i,k}\cdot {\mathrm{Recall}}_{c,j,i,k}}{{\mathrm{Precision}}_{c,j,i,k}+{\mathrm{Recall}}_{c,j,i,k}}$30:           **end for**31:        **end for**32:        **Calculate the average of each metric over the five folds for model $M_{j}$ on dataset $D_{i}$** using Equation (13)33:        **Normalize metrics for Model $M_{j}$ on dataset $D_{i}$** using Equations (14) and (15)34:        **Calculate the weighted average of normalized metrics for model $M_{j}$ on dataset $D_{i}$** using Equation (16)35:    **end for**36:**end for**37:**Model Selection:**38:Calculate the overall score for each model $M_{j}$ by averaging the scores across all datasets $D_{1},D_{2},D_{3}$ using Equation (17)39:Select the best model $M^{*}$ based on the highest final score using Equation (18)40:**Output:** Return the best model $M^{*}$ based on the highest overall score across all datasets.

In light of the challenges inherent in CE cyst classification, our study employs a systematic evaluation of multiple DL models under various hyperparameter configurations. These models were trained and tested on datasets specific to CT, US, and MRI modalities, with a strong focus on ensuring their generalization capability across small and imbalanced datasets. To achieve this, we adopted a comprehensive evaluation framework incorporating a wide range of metrics, including accuracy, sensitivity, specificity, and AUC–ROC, to assess the models’ effectiveness in real-world clinical scenarios. The subsequent section details the experimental setup and results, providing a thorough analysis of model performance across modalities and metrics. This analysis offers critical insights into the trade-offs and strengths of each approach, ultimately guiding the selection of the most robust and reliable model for CE cyst classification.

## 6. Experimental Results

The experimental evaluation was conducted to validate the proposed solution’s efficacy in classifying CE cysts into active, transitional, and inactive stages. This section provides a comprehensive overview of the models’ performance across three imaging modalities (CT, US, and MRI), focusing on the critical metrics outlined earlier. By systematically analyzing these results, we aim to identify the optimal model that balances diagnostic accuracy with clinical applicability.

### 6.1. Experimental Setup

A High-Performance Computing (HPC) server with the following specifications was used for all experiments in this study: an Intel Xeon W-2133 processor, 40 GB of RAM, and an NVIDIA GTX 1080 (NVIDIA A100 80GB PCIe) Graphics Processing Unit (GPU). The server ran on Linux, with GPU management handled by the CUDA Toolkit 12.3. The setup included a four-node DELL EMC PowerEdge R740 rack server. We used Python 3.11.0rc1, Keras 3.8.0, and TensorFlow 2.17.0 as the backend to train DL models.

Evaluation metrics in machine learning often vary not only in their scale but also in their interpretation, making direct comparison and aggregation challenging. For instance, accuracy values typically range from 0% to 100%, AUC–ROC values range from 0 to 1 (where higher values indicate better performance), and loss values can range from 0 to 10 or more, with lower values indicating better performance. This difference in interpretation, especially for metrics like loss, complicates the process of evaluating models holistically.

To address this issue, we implemented a normalization process that standardizes all metrics to a consistent range (e.g., 0 to 1) and interprets higher values as indicative of better performance. This unified framework facilitates meaningful comparison and aggregation of metrics, ensuring consistency in evaluation across diverse metrics. Therefore, each table summarizes both evaluation metrics (Avg.μ) and their normalized values (Norm.μ), along with a composite score (Score) that ranks the overall performance of each model. The experimental results in Table 10, Table 11 and Table 12 compare the performance of 10 model architectures on CT, US, and MRI datasets. These architectures include Inception ($M_{1}$), ResNet ($M_{2}$), VGG16 ($M_{3}$), VGG19 ($M_{4}$), CNN ($M_{5}$), FNN ($M_{6}$), Inception+ResNet ($M_{7}$), CNN+ResNet ($M_{8}$), MobileNet ($M_{9}$), and ViT ($M_{10}$) as denoted.

The tables present the performance evaluation of different models across multiple datasets, with each row representing a model and columns detailing various performance metrics, including the Score value, which serves as the primary criterion for selecting the best model. For each dataset, the model with the highest **Score** is highlighted in yellow, indicating the best-performing model. This visual distinction facilitates an easy comparison of model performance across datasets and helps identify the most effective model for each case. Based on the analysis, $M_{8}$ (Table 10) for the CT dataset, $M_{7}$ (Table 11) for the US dataset, $M_{8}$ (Table 12) for the MRI dataset outperforms others, achieving the highest Score consistently. This superiority can be attributed to its ability to balance key performance metrics, demonstrating robustness and generalizability across different datasets, making it the most suitable model for this task.

### 6.2. Experimental Results on CT Dataset

CNN+Resnet ($M_{8}$) achieves the highest composite score of 0.990, making it the most effective model for the CT dataset as summarized in Table 10. Its high performance is reflected in all evaluation metrics, including accuracy (97.55%), precision (96.5%), and AUC–ROC (99.4%).

Inception+ResNet ($M_{7}$) and Inception ($M_{1}$) are close contenders with composite scores of 0.988 and 0.983, respectively. These models consistently deliver high accuracy and balanced sensitivity values, making them reliable for medical applications. Thus, MobileNet ($M_{9}$) and ViT ($M_{10}$) achieve moderate performance with composite scores of 0.537 and 0.631, respectively. While their accuracy and sensitivity are reasonable, their lower weighted F1 scores and normalized metrics limit their suitability for the dataset. CNN ($M_{5}$) and FNN ($M_{6}$) perform poorly, with composite scores of 0.080 and 0.062, respectively. These models struggle to achieve acceptable levels of accuracy, precision, and recall, making them unsuitable for the given task.

Inception+ResNet and CNN+ResNet significantly outperform single models, with CNN+ResNet achieving the highest accuracy (97.55%). Hybrid models CNN+ResNet ($M_{8}$) and Inception+ResNet ($M_{7}$) excel in balancing these metrics, making them highly suitable for clinical tasks where both false positives and false negatives must be minimized, due to their architectural ability to learn hierarchical and diverse features. They have architectures with advanced feature extraction capabilities, such as ResNet’s residual connections or Inception’s multi-scale filters, and tend to achieve higher accuracy. These designs enhance the model’s ability to capture complex patterns, reducing misclassifications. These models combine the strengths of complementary architectures (e.g., Inception’s multi-scale feature extraction and ResNet’s skip connections), enabling them to capture a broader range of features. This synergy leads to better generalization and robustness, particularly in structured and complex datasets like CT scans.

**Figure 4:**
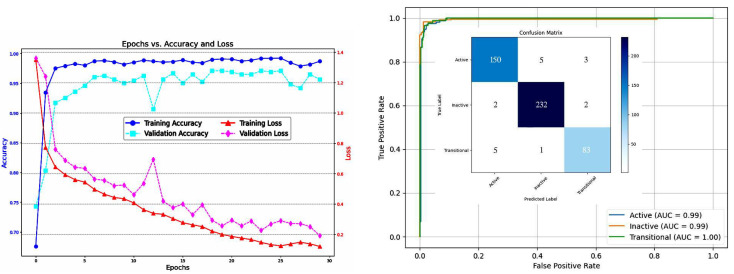
Dataset 1—CT, CNN+ResNet model’s results.

In Figure 4, to explain the confusion matrix and classification report of the best model (CNN+ResNet) in detail, these aspects should be covered. First, the model performs well for all three classes with a high AUC (0.99). This indicates the model reliably identifies all instances while minimizing false positives and negatives.

Stratified K-fold cross-validation may lead to slight variations of ±1 in the number of cases per class in the test set as shown in the confusion matrix in Figure 4. This occurs because, although the method preserves overall class distribution, the exact division of samples across folds may result in small differences, especially when class sizes are not perfectly divisible by the number of folds. These minor variations do not significantly affect the overall evaluation, as shown in Table 11.

The model exhibits strong performance across both training and validation phases over the course of 30 epochs. Training accuracy approaches 100%, indicating effective learning and optimization of the training data, while the steady decline in training loss further supports efficient model convergence. Validation accuracy remains consistently high with minor fluctuations, suggesting the model generalizes well to unseen data. Additionally, although the validation loss shows some oscillation, it decreases substantially over time, reflecting ongoing improvements in minimizing errors on the validation set. Overall, these results highlight the model’s robust learning ability and good generalization, with potential for further refinement through additional techniques to smooth out validation performance.

### 6.3. Experimental Results on US Dataset

The experimental results on the US dataset reveal that hybrid models such as Inception+ResNet and CNN+ResNet outperform single models according to score (0.997 and 0.980), achieving high weighted average accuracy (94.65%, 93.99%), precision (95.6%, 93.3%), recall, and F1 score due to their ability to capture complex features and patterns in Table 11.

Inception+ResNet ($M_{7}$) also performs well, benefitting from the combination of multiple model strengths. In contrast, models like VGG16, VGG19, MobileNet, and FNN show poor results, likely due to the small dataset size and their limitations in capturing relevant US image features. Overall, hybrid models significantly enhance performance on small, augmented datasets, highlighting their superior ability to generalize and effectively process medical images.

**Figure 5:**
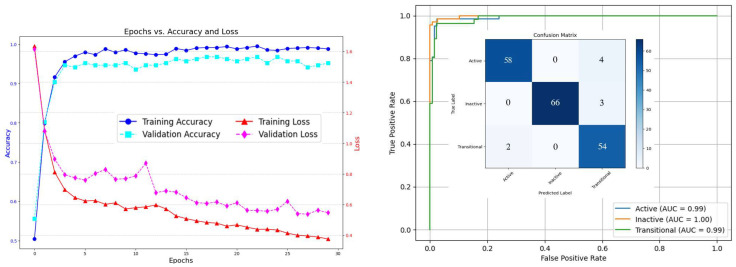
Dataset 2—US, Inception+ResNet model’s results.

In Figure 5, we included Inception+ResNet ($M_{7}$ model’s results on the US dataset). Here, the model achieves a high AUC–ROC score (over 99.0%), indicating strong overall performance. We also address class imbalance by applying SMOTE, which can be effective in improving model performance, particularly for ResNet and Inception architectures. Both models are known for their ability to capture complex patterns and features, which benefit from a balanced dataset. The use of SMOTE in combination with ResNet or Inception enhances performance by better handling class imbalance, resulting in improved generalization and more accurate predictions across all classes—active, transitional, and inactive. Additionally, we evaluated all models on the original imbalanced dataset, where the model performs exceptionally well in classifying active and inactive cases, achieving near-perfect precision and recall. While precision and recall for the transitional class remain strong, they are slightly lower compared with the other two classes. This discrepancy is due to the best model struggling with the transitional class, misclassifying three instances as inactive, suggesting that the distinguishing features between transitional and inactive are less distinct or more challenging for the model to learn.

The model demonstrates excellent performance in training on the US dataset, with the training accuracy (represented by the blue line with circles) reaching nearly 100% and remaining steady across all epochs. This indicates that the model is highly effective at learning the patterns in the training data. Additionally, the training loss (shown by the red line with triangles) steadily decreases, suggesting that the model is successfully minimizing errors and improving its fit to the training set. Validation accuracy (the cyan line with squares) exhibits a rapid increase during the initial epochs, followed by a stabilization at a high level, which indicates strong generalization to unseen data. This consistent high performance on the validation set confirms that the model is not overfitting and can effectively apply learned features to new examples. Furthermore, the validation loss (magenta line with diamonds) shows a significant decrease over the epochs, with minor fluctuations, indicating that the model is refining its predictions and reducing errors on the validation set. The rapid learning observed in the first few epochs, reflected in both accuracy and loss metrics, further highlights the model’s ability to quickly adapt to the data and learn the relevant features. Overall, the results demonstrate that the model is performing exceptionally well, with strong generalization to unseen data and effective error reduction. Minor adjustments may help stabilize the validation performance even further, but the current results indicate a robust and successful model.

### 6.4. Experimental Results on MRI Dataset

On the MRI dataset, the experimental results show varying performance across the models, with CNN+ResNet and Inception+ResNet achieving perfect scores in accuracy (100%), precision (1.00), recall (1.00), and F1 score (1.00), demonstrating flawless classification and ideal for tasks requiring minimal false positives and false negatives as shown in Table 12. CNN+ResNet and Inception+ResNet also perform well, with accuracy above 90%, high precision and recall (0.95 and above), and well-balanced F1 scores. ResNet50 shows strong results with an accuracy of 81%, precision of 0.86, and recall of 0.81, but could benefit from slight improvements. Inception delivers moderate performance, while VGG16, VGG19, and FNN show poor results with low accuracy (around 70%) and poor balance between precision and recall, indicating a need for significant improvement or tuning.

Hybrid models like Inception+ResNet and CNN+ResNet consistently outperform single models with scores above 0.95 across all metrics due to the combination of strengths from each individual model. By integrating the unique capabilities of each model, these hybrid architectures are able to capture a broader and more diverse set of features and patterns in the data. This fusion helps mitigate the weaknesses of each model on its own, improving their generalization ability and making them more robust to overfitting. Additionally, hybrid models are particularly effective in handling small or complex datasets, as they learn complementary representations of the data, which is essential in challenging tasks like medical image classification. As a result, these hybrid models achieve superior performance, offering a balanced and comprehensive approach to feature extraction and classification.

VGG16 and VGG19 are deep CNN architectures that contain many layers and a large number of parameters. These models were originally designed to perform well on large and diverse datasets like ImageNet, which contains millions of images from a wide variety of categories. However, when applied to small or highly specialized datasets like the MRI images in this experiment, they can easily overfit, meaning they memorize the limited examples in the training data instead of learning generalizable patterns. Inception also uses a more complex design by employing multiple filter sizes at different stages of the network. This makes it suitable for a wide variety of tasks but could be inefficient for a small dataset where a simpler architecture might perform better. Inception and VGG models were trained on a vast variety of images from general categories (animals, objects, etc.), while MRI images are a very specific type of medical data. This results in a domain shift, where the features learned by the model on general images do not transfer well to MRI images. For example, MRI images have unique characteristics like noise patterns, image artifacts, and anatomical structures that are not present in the images that these models were initially trained on. Given these factors, Inception, VGG16, and VGG19 may struggle to perform well on small and domain-specific datasets like MRI images, especially when compared with lighter models such as MobileNet and ViT, which are more efficient at generalizing with smaller amounts of data.

Data augmentation likely had a positive impact on the model’s performance, especially in increasing the number of images from 42 to 4200. Augmenting the dataset helps improve the model’s generalization by introducing variations in the training images, which allows the model to learn more robust and diverse features.

**Figure 6:**
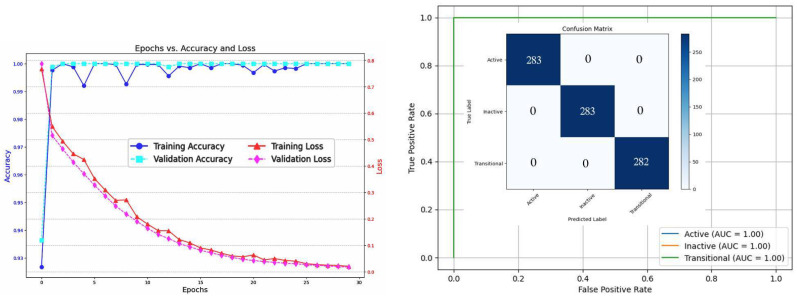
Dataset 3—MRI, CNN+ResNet model’s results.

From Figure 6, the MRI training and validation accuracies and losses curve indicates that the model’s performance is good, as evidenced by several key observations. Both training and validation accuracies exceed 99%, suggesting that the model performs very well on the given data, while both training and validation losses approach 0, indicating effective error minimization. Furthermore, there is a minimal gap between training and validation accuracy/loss, which demonstrates that the model generalizes well and avoids overfitting. However, it is important to consider the quality and diversity of the dataset, as high accuracy might arise from overly similar training and validation sets, potentially leading to lower real-world performance. Additionally, the relatively small size of the dataset may limit the model’s generalization to larger, more diverse datasets. If the dataset is well-prepared and properly split, the results indicate a well-performing model. Nonetheless, testing the model in real-world scenarios is crucial to confirm its reliability and robustness. In the confusion matrix and AUC–ROC curve, the sensitivity (or recall) of the CNN+ResNet model on the balanced MRI dataset demonstrates excellent performance in classifying cystic lesions in medical images, particularly in the context of CE cyst classification. Specifically, the model achieves perfect sensitivity of 1.00 for all categories without any false negatives. This is crucial for medical image classification, as detecting these key stages of cyst development is essential for accurate diagnosis and treatment planning. Nevertheless, the overall high sensitivity in the balanced MRI dataset underscores the model’s ability to effectively capture critical features related to cyst stages, contributing to accurate CE cyst classification in medical imaging.

Table 13 presents the performance and ranking of ten deep learning models based on their scores for detecting cysts using three different imaging modalities: CT, ultrasound (US), and MRI. The columns show the scores for each modality, the final aggregated score (FinalScore), and the model’s rank based on overall evaluation performance. The models are ranked from highest to lowest FinalScore, where the top-performing model is ranked I and the lowest-ranked model is ranked X. The table is essential for comparing the effectiveness of various models in medical imaging applications, especially for cyst detection. $M_{7}$—Inception+ResNeT achieves the highest FinalScore of 0.994, with near-perfect performance across all imaging types (CT, US, MRI), making it the top model in this evaluation. So $M_{7}$ should be the best model as $M^{*}$. Then, $M_{8}$—CNN+ResNeT ranks second with a FinalScore of 0.986, slightly lower than $M_{7}$ but still showing high consistency and strong performance across modalities. The model’s flexibility to adapt to various imaging modalities is a significant advantage, ensuring its robustness when deployed in real clinical scenarios, where one modality may be preferred over others. Cysts in CE images may present challenges due to factors like varying contrast levels, surrounding tissues, or artifacts caused by the imaging process. The advanced capabilities of Inception+ResNeT in handling complex images with varying levels of detail make it well suited for overcoming these challenges. It can accurately detect cysts even when their appearance is subtle or when the image quality is not ideal. In clinical settings, cyst classification requires a model that can handle a variety of image types efficiently. Inception+ResNeT’s high adaptability and strong generalization abilities make it suitable for clinical applications where CT and US are more commonly available than MRI. This allows clinicians to rely on this model for cyst diagnosis even in resource-limited settings.

In conclusion, while artificial and synthetic datasets can be useful for initial testing and model development, they often fail to capture the full complexity and variability of real-world data. The use of real-world datasets, coupled with data augmentation techniques such as US and MRI datasets, offers a more comprehensive and reliable approach for evaluating ML models. Real data reflects the inherent variability present in clinical and environmental conditions, providing a more accurate representation of how models will perform in practical applications. By augmenting these real datasets, we can enhance the diversity and richness of the data, enabling the model to generalize better across a wider range of scenarios. This approach leads to improved model performance, as evidenced by more robust and reliable results, ultimately contributing to more effective and real-world applicable solutions.

In the medical field, sensitivity, specificity, and F1 score are crucial metrics for evaluating the performance of diagnostic models. Medical diagnoses often involve critical decision-making, where false negatives (missed diagnoses) can have severe consequences, making sensitivity a priority in early disease detection. On the other hand, excessive false positives (overdiagnosis) can overwhelm healthcare systems and patients, underscoring the importance of specificity in minimizing unnecessary interventions. The F1 score provides a balanced evaluation, particularly in scenarios where both types of errors—false negatives and false positives—are undesirable, ensuring the model performs well across various classification aspects. This is especially valuable when dealing with imbalanced datasets. We compare the performance of the best models using these metrics, as summarized in Table 14.

- For MRI, all three model architectures (CNN+ResNet, Inception+ResNet, Inception) consistently achieve perfect scores (sensitivity, specificity, F1 score = 1.000) across all stages (active, transitional, inactive).
- For CT, Inception+ResNet generally outperforms the other architectures, particularly for the transitional stage, with high sensitivity (0.910) and specificity (0.994).
- For US, CNN+ResNet demonstrates strong performance, especially in the inactive stage, where it achieves specificity = 0.991 and F1 score = 0.977.

Overall, MRI consistently delivers the best results, with all metrics reaching perfect or near-perfect values across all models and stages. US, however, tends to show slightly lower performance compared with MRI and CT, particularly for the Inception and Inception+ResNet models in the active and transitional stages.

Table 15 presents a comparison of various deep learning model architectures, their settings, and their performance across CT, US, and MRI datasets. The models range from traditional CNNs to more advanced architectures, including VGG16, VGG19, Inception, ResNet50, and ViT, with varying numbers of trained and trainable parameters. The number of trainable parameters spans from 23,907,779 (FNN) to 86,415,592 (ViT), with deeper models generally having more parameters. Pre-trained models, such as VGG16 and ResNet50, demonstrate superior performance across all datasets, especially on MRI, where they achieve near 100% accuracy. Hyperparameters such as learning rate (0.00001 or 0.001), dropout rate (0.4–0.6), and epochs (30) are consistent across models to ensure stable training. Regularization techniques, like L2 regularization, are applied to minimize overfitting. MobileNet, with fewer parameters (38,523), shows lower performance on the US dataset, while the custom FNN architecture performs moderately across all datasets. Overall, deeper, pre-trained models with higher parameter counts tend to deliver better performance, particularly on complex datasets like MRI, while lighter models, though computationally more efficient, may struggle with certain datasets. These results strongly support our hypothesis that reducing the number of trainable parameters can enhance model performance. By simplifying the model and limiting its complexity, we effectively mitigate the risk of overfitting, which arises when a model becomes overly specialized to the training data and struggles to generalize to new, unseen examples. This reduction in the number of parameters results in more efficient and resilient models, which are better equipped to make accurate predictions, particularly when working with smaller image datasets. Consequently, the model’s robustness and ability to generalize improve, making it more reliable for real-world applications.

The results obtained from these models are reasonable given the dataset size of 1000 to 4000 images after augmentation and sampling. While deep learning models generally require large datasets to generalize effectively, the application of data augmentation and oversampling techniques, such as SMOTE, helps mitigate overfitting by artificially increasing the diversity and size of the training data. With this dataset size, the performance of models like VGG16 and ResNet50, especially when pre-trained, should be satisfactory as they have the capacity to generalize from the augmented data. For datasets ranging from 1000 to 4000 images, particularly after augmentation, complex models like VGG16, VGG19, ResNet50, and Inception should perform well, especially if the models are fine-tuned effectively. Lighter models such as MobileNet may still yield decent results but could face challenges in capturing the complexities of medical image classification, especially with smaller datasets or certain modalities like US. However, depending on class balance and task complexity, it is crucial to use cross-validation to prevent overfitting and assess the model’s generalization to unseen data. Additionally, experimenting with different architectures is essential to find the optimal balance between model complexity and dataset size for the specific application.

In Table 16, we compare the results of our best model with the gold standard of diagnostic tools for CE. Non-imaging diagnostic tools play a crucial role in cyst classification, particularly in complementing imaging methods. Serological tests like ELISA (sensitivity 80–94%, specificity 85–95%) detect antibodies against Echinococcus antigens but have reduced sensitivity for calcified or lung cysts. Immunoblot offers greater accuracy (sensitivity 88–96%, specificity 97–99%) by confirming specific antigens such as AgB or Ag5. The definitive diagnosis can be achieved through histopathology (100% sensitivity and specificity) by identifying parasite structures in surgically resected cysts or biopsies. PCR (sensitivity and specificity 100%) provides species-level identification by detecting Echinococcus DNA in cyst fluid or tissue, making it a molecular gold standard for precise diagnosis. These tools are essential for confirming diagnoses, particularly in complex cases.

The best AI model for cyst classification demonstrates exceptional diagnostic performance, with sensitivity scores of 96.0% on CT, 95.4% on ultrasound (US), and 100% on MRI, and specificity scores of 98.1% on CT, 97.8% on US, and 100% on MRI. This performance, closely matching or exceeding human ground truth accuracy, highlights AI’s potential as a reliable diagnostic tool. For instance, AI achieved an overall sensitivity of 97.13% across three datasets, outperforming many traditional diagnostic tools in both sensitivity and specificity.

## 7. Conclusions

In conclusion, this study provides a comprehensive evaluation of 10 machine learning (ML) algorithms across three distinct imaging modalities—CT, US, and MRI—designed to classify cystic echinococcosis (CE) cysts into three stages: active, inactive, and transitional. The performance of these algorithms was assessed using weighted average accuracy, precision, recall, and F1 score, yielding significant insights into their strengths and limitations. Among the evaluated models, CNN+ResNet consistently outperformed others across all datasets, achieving accuracies ranging from 94.65% to 100%. This model demonstrated exceptional capability in capturing complex features and patterns within the image data, underscoring the potential of hybrid architectures in medical image classification. Moreover, the strong performance of hybrid models like CNN+ResNet and Inception+ResNet highlights the advantages of leveraging multiple architectural strengths to enhance predictive accuracy. Additionally, the study emphasizes the critical role of dataset characteristics—including image quality, diversity, and size—in shaping model performance. By incorporating data augmentation and oversampling techniques to address the challenges of limited medical data, this study ensured improved model performance, showcasing the robustness of these approaches. These findings position hybrid models as promising solutions for real-world healthcare applications requiring efficient and accurate diagnostic support.

## 8. Patents

Registered and certified by Order No. A/97 of the Director of the Intellectual Property Office of Mongolia dated 26 August 2024. Registration number: 16003.

## Figures and Tables

**Table 1:** Gharbi classification of CE.

Type	Morphological Characteristics
I	Purely liquid-filled cyst, regular wall and internal, anechoic (clear)
II	Liquid cyst with floating membranes, detached germinal layer (water-lily sign)
III	Multivesicular, presence of daughter cyst (honeycomb pattern), liquid + solid components
IV	Heterogeneous cyst, no daughter vesicules, predominantly solid, no clear liquid component
V	Cyst with partially or completely calcified walls, strong posterior shadowing, dead cyst

**Table 2:** WHO-IWGE classification of CE.

Type	Characteristics	Pathophysiological Stage
CE1	Uniloculer, anechoic cyst with double line sign	Active
CE2	Multiseptated “rosette-like” “honeycomb pattern” cyst	Active
CE3a	Cyst with detached membrane (water-lily sign)	Transitional
CE3b	Daughter cysts in solid matrix	Transitional
CE4	Heterogeneous cyst, no daughter vesicules	Inactive
CE5	Solid matrix with calcified wall	Inactive

**Table 3:** WHO-IWGE classification system and imaging modalities.

...	US	MRI	CT
**CE1**	Anechoic cyst with “double-line sign” (pericyst and endocyst).	T1: Hypointense; T2: Hyperintense; clear fluid content.	Hypodense, well-defined lesion with a thin wall.
**CE2**	Multiseptated “honeycomb” structure with daughter cysts.	T2: Hyperintense daughter cysts; “rosette” or “wheel-spoke” appearance.	Multilocular lesion with daughter cysts; septations visible.
**CE3a**	Detached membranes (“water-lily sign”) visible within fluid.	T2: Hypointense floating membranes in hyperintense fluid.	Floating membranes in a low-density cyst.
**CE3b**	Daughter cysts with degenerating content; mixed echogenicity.	T2: Mixed signal intensity; partial degeneration visible.	Mixed density cyst with septations and daughter cysts.
**CE4**	Heterogeneous echotexture; solid appearance without clear fluid.	T1/T2: Low signal intensity due to necrotic tissue.	Low-density lesion with some solid components; no enhancement.
**CE5**	Thick calcified wall with strong posterior shadowing.	Signal void (calcifications) on all sequences.	Dense calcified rim; no active content.

**Table 4:** Overview of ML models for identifying stage of CE.

№	Methods	Structure	Application	Adv./Disadv.
$M_{1}$	Inception	Wider network design using “inception modules” that can capture multi-scale features.	High performance on complex tasks with multiple objects/scales.	Very powerful but computationally demanding
$M_{2}$	ResNet50	ResNet-50 has 50 layers, making it a deep architecture. This depth enables it to capture intricate features from complex data, enhancing performance on tasks like image recognition and classification.	It also serves as a robust backbone for transfer learning, where it’s pre-trained on a large dataset (e.g., ImageNet) and fine-tuned on specific tasks.	While ResNet-50 offers high performance, it requires more memory and processing power compared with simpler models due to its depth.
$M_{3}$, $M_{4}$	VGG16 & VGG19	Deep networks with 16 or 19 layers, enabling improved feature extraction.	Suitable for complex image processing tasks but resource-intensive due to high depth.	High accuracy but requires more computational power for training and deployment.
$M_{5}$	CNN	Convolutional layers, pooling layers, and fully connected layers. Varies in depth depending on the task.	Often used for simpler tasks due to lower depth, which limits performance for more complex tasks.	Relatively lightweight, but lacks advanced depth.
$M_{6}$	FNN	Simplest architecture of ANN without convolutional layers, often fully connected.	Basic tasks where speed is prioritized over accuracy.	Fast, but lower performance on complex task
$M_{7}$	Inception + Resnet	Combination of Inception’s multi-scale features and ResNet’s residual connections.	Useful for capturing complex and large-scale patterns	High-performing, flexible architecture that balances depth and width.
$M_{8}$	CNN + Resnet	This hybrid approach is often used to balance the strengths of both architectures: the simplicity and efficiency of simple CNNs and the enhanced depth and stability of ResNet.	The CNN + ResNet architecture is ideal for applications that require both robustness in learning complex patterns and efficient feature extraction.	The resource consumption of a CNN + ResNet depends on various factors, including the architecture’s depth, input data size, hardware, and optimization settings.
$M_{9}$	MobileNet	Optimized for mobile and embedded devices using depthwise separable convolutions.	Lightweight and faster, suitable for real-time applications.	Balanced, trading off some accuracy for reduced computational load.
$M_{10}$	ViT	Transformer-based, processes images as sequences of patches.	State-of-the-art for image classification, due to its unique handling of images as sequential data.	High performance and flexibility, often surpassing CNN-based models in certain benchmarks, though resource-intensive.

**Table 5:** MRI images from literature review.

		P1 [28]	P2 [29]	P3 [30]	P4 [31]	P5 [32]	P6 [33]	P7 [34]	P8 [35]	P9 [36]	Total
Active	CE1	1	2	1		1	1		3		14
	CE2	1	2			1	1				
Transitional	CE3a		2			1	1	6			14
	CE3b		2			1	1				
Inactive	CE4		2		3	1	1			3	14
	CE5		2			1	1				

**Table 6:** Original size of experimental CT, US, and MRI datasets.

	Active	Transitional	Inactive	Total
$D_{1}$—CT images	792	444	1180	2416
$D_{2}$—US images	29	25	31	85
$D_{3}$—MRI images	14	14	14	42

**Table 7:** $D_{1}$: Summary of CT image distribution across original, test, and training sets.

	Active	Transitional	Inactive	Total
Original	792	444	1180	2416
Test (20%)	158	89	236	483
Train (80%)	634	355	944	1933
Sampled (Train)	944	944	944	2832

**Table 8:** $D_{2}$: US images were augmented, divided into training and testing subsets using an 80:20 split, and balanced through oversampling.

US	Active	Transitional	Inactive	Total
Original	29	25	31	85
Augmented	319	275	341	935
Test (20%)	62	56	69	187
Train (80%)	256	219	273	748
Sampled	273	273	273	819

**Table 9:** $D_{3}$: MRI images were augmented and divided into training and testing sets.

	Active	Transitional	Inactive	Total
Original	14	14	14	42
Augmented	1414	1414	1414	4242
Test (20%)	283	283	282	848
Train (80%)	1131	1131	1131	3393

**Table 10:** Experimental results of models on CT dataset ($D_{1}$).

	Evaluation Metrics (Avg.μ)									Normalized Metrics (Norm.μ)									Score
**M**	Avr.A	Avg.L	Avg.P	Avg.R	Mean.F1	Avg.AUC	Avr.S	Avr.Sp	Avg.WF	Norm.A	Norm.L	Norm.P	Norm.R	Norm.F1	Norm.AUC	Norm.S	Norm.Sp	Norm.WF	
$M_{1}$	97.36%	0.325	0.964	0.964	0.968	0.992	0.964	0.983	0.968	0.996	0.91	0.996	0.998	0.999	0.996	1.000	1.000	0.964	0.983
$M_{2}$	97.26%	0.251	0.967	0.965	0.969	0.993	0.962	0.962	0.969	0.994	0.95	1.000	1.000	1.000	0.007	0.997	0.933	1.000	0.930
$M_{3}$	78.75%	0.445	0.777	0.831	0.777	0.895	0.831	0.915	0.778	0.587	0.84	0.749	0.788	0.726	0.799	0.789	0.785	0.522	0.926
$M_{4}$	84.09%	1.939	0.804	0.753	0.787	0.921	0.753	0.880	0.787	0.704	0.00	0.785	0.665	0.740	0.852	0.665	0.674	0.524	0.603
$M_{5}$	52.05%	1.075	0.212	0.366	0.286	0.523	0.366	0.679	0.286	0.000	0.49	0.004	0.051	0.024	0.047	0.051	0.038	0.017	0.080
$M_{6}$	65.07%	1.098	0.209	0.334	0.269	0.500	0.334	0.667	0.269	0.286	0.48	0.000	0.000	0.000	0.000	0.000	0.000	0.000	0.062
$M_{7}$	96.78%	0.169	0.959	0.961	0.964	0.994	0.961	0.982	0.964	0.983	1.00	0.989	0.994	0.993	1.000	0.994	0.997	0.961	0.988
$M_{8}$	97.55%	0.244	0.965	0.960	0.966	0.994	0.960	0.981	0.966	1.000	0.960	0.997	0.992	0.996	1.000	0.994	0.994	0.963	**0.990**
$M_{9}$	79.67%	0.891	0.707	0.601	0.652	0.817	0.601	0.802	0.652	0.607	0.59	0.656	0.424	0.547	0.641	0.424	0.427	0.459	0.537
$M_{10}$	89.67%	0.891	0.818	0.701	0.754	0.957	0.702	0.882	0.756	0.827	0.59	0.803	0.582	0.693	0.925	0.585	0.681	0.503	0.631

**Bold** Score values highlight the highest Score in each column, while yellow-highlighted rows indicate the best-performing models for each dataset.

**Table 11:** Experimental results of models on US dataset $D_{2}$.

	Evaluation Metrics (Avg.μ)									Normalized Metrics (Norm.μ)									Score
**M**	Avr.A	Avg.L	Avg.P	Avg.R	Mean.F1	Avg.AUC	Avr.S	Avr.Sp	Avg.WF	Norm.A	Norm.L	Norm.P	Norm.R	Norm.F1	Norm.AUC	Norm.S	Norm.Sp	Norm.WF	
$M_{1}$	93.64%	0.977	0.933	0.932	0.933	0.990	0.311	0.967	0.934	0.981	0.745	0.722	0.962	0.968	0.991	0.000	0.963	0.954	0.888
$M_{2}$	88.47%	1.258	0.912	0.899	0.900	0.970	0.899	0.951	0.900	0.889	0.567	0.941	0.907	0.920	0.949	0.914	0.910	0.906	0.871
$M_{3}$	67.89%	0.966	0.727	0.700	0.698	0.853	0.700	0.851	0.698	0.527	0.752	0.692	0.569	0.634	0.702	0.605	0.574	0.623	0.626
$M_{4}$	79.36%	2.151	0.766	0.760	0.763	0.906	0.760	0.822	0.763	0.730	0.000	0.745	0.671	0.726	0.814	0.698	0.477	0.714	0.602
$M_{5}$	52.05%	1.705	0.211	0.365	0.286	0.522	0.366	0679	0.286	0.246	0.283	0.000	0.000	0.047	0.000	0.085	0.000	0.046	0.074
$M_{6}$	38.18%	1.085	0.260	0.378	0.253	0.550	0.379	0.691	0.253	0.000	0.676	0.066	0.022	0.000	0.059	0.106	0.040	0.000	0.107
$M_{7}$	94.65%	0.575	0.956	0.954	0.956	0.994	0.954	0.978	0.956	1.000	1.000	1.000	1.000	1.000	1.000	1.000	1.000	0.983	**0.997**
$M_{8}$	93.99%	0.639	0.937	0.936	0.937	0.993	0.936	0.969	0.967	0.988	0.959	0.974	0.970	0.974	0.998	0.973	0.970	1.000	0.980
$M_{9}$	60.35%	1.266	0.664	0.641	0.642	0.837	0.642	0.824	0.642	0.392	0.562	0.608	0.469	0.554	0.667	0.514	0.485	0.545	0.612
$M_{10}$	91.07%	0.798	0.890	0.905	0.895	0.930	0.852	0.758	0.865	0.936	0.859	0.912	0.916	0.914	0.865	0.841	0.264	0.857	0.745

**Bold** Score values highlight the highest Score in each column, while yellow-highlighted rows indicate the best-performing models for each dataset.

**Table 12:** Experimental results of models on MRI dataset.

	Evaluation Metrics (Avg.μ)									Normalized Metrics (Norm.μ)									Score
**M**	Avr.A	Avg.L	Avg.P	Avg.R	Mean.F1	Avg.AUC	Avr.S	Avr.Sp	Avg.WF	Norm.A	Norm.L	Norm.P	Norm.R	Norm.F1	Norm.AUC	Norm.S	Norm.Sp	Norm.WF	
$M_{1}$	100.00%	0.207	1.000	1.000	1.000	1.000	1.000	1.000	1.000	1.000	0.896	1.000	1.000	1.000	1.000	1.000	1.000	1.000	0.989
$M_{2}$	99.99%	0.076	1.000	1.000	1.000	1.000	1.000	1.000	1.000	0.999	0.994	1.000	1.000	1.000	1.000	1.000	1.000	1.000	0.949
$M_{3}$	85.80%	0.866	0.896	0.872	0.873	0.986	0.872	0.936	0.873	0.857	0.409	0.600	0.591	0.665	0.933	0.592	0.739	0.665	0.629
$M_{4}$	99.88%	1.418	0.997	0.996	0.998	0.999	0.997	0.997	0.994	0.998	0.000	0.988	0.987	0.994	0.996	0.991	0.988	0.983	0.891
$M_{5}$	61.68%	0.534	0.740	0.687	0.621	0.794	0.686	0.843	0.621	0.000	0.655	0.000	0.000	0.000	0.000	0.000	0.362	0.000	0.138
$M_{6}$	77.42%	0.413	0.777	0.711	0.671	0.853	0.719	0.855	0.671	0.255	0.746	0.142	0.077	0.132	0.287	0.105	0.410	0.132	0.263
$M_{7}$	99.99%	0.068	0.999	0.999	0.999	1.000	0.999	0.999	0.999	0.999	1.000	0.996	0.997	1.000	1.000	0.997	0.997	0.997	0.9987
$M_{8}$	100.00%	0.075	1.000	1.000	1.000	1.000	1.000	1.000	1.000	1.000	0.994	1.000	1.000	1.000	1.000	1.000	1.000	1.000	**0.9994**
$M_{9}$	90.86%	0.492	0.940	0.939	0.939	0.993	0.936	0.969	0.939	0.572	0.686	0.769	0.806	0.839	0.967	0.796	0.874	0.839	0.822
$M_{10}$	78.96%	0.500	0.775	0.698	0.775	0.850	0.790	0.754	0.755	0.268	0.680	0.135	0.035	0.406	0.272	0.331	0.000	0.354	0.274

**Bold** Score values highlight the highest Score in each column, while yellow-highlighted rows indicate the best-performing models for each dataset.

**Table 13:** Ranking of models for cyst classification based on US, CT, and MRI scores.

Model	Score (CT)	Score (US)	Score (MRI)	FinalScore	Rank
$M_{1}$—Inception	0.964	0.888	0.989	0.947	III
$M_{2}$—ResNet50	0.930	0.871	0.949	0.917	IV
$M_{3}$—VGG16	0.522	0.626	0.629	0.592	VII
$M_{4}$—VGG19	0.603	0.602	0.891	0.699	V
$M_{5}$—CNN	0.080	0.074	0.138	0.097	X
$M_{6}$—FNN	0.062	0.107	0.263	0.144	IX
$M_{7}$—Inception+ResNeT	0.988	0.997	0.9987	0.994	I
$M_{8}$—CNN+ResNeT	0.990	0.980	0.9994	0.986	II
$M_{9}$—MobilNeT	0.537	0.612	0.822	0.657	VI
$M_{10}$—ViT	0.631	0.745	0.274	0.550	VIII

**Table 14:** Comparison of diagnostic model performance across modalities (MRI, CT, US) based on sensitivity, specificity, and F1 score at different stages (active, transitional, inactive).

	Best Models	CNN+ResNet			Inception+ResNet			Inception		
Metrics	Modularities	CT	US	MRI	CT	US	MRI	CT	US	MRI
	Stages									
**Sensitivity**	**Active**	0.955	0.951	1	0.968	0.935	1	0.962	0.870	0.962
	**Transitional**	0.988	0.964	1	0.910	0.964	0.989	0.943	0.964	0.943
	**Inactive**	0.991	0.970	1	0.974	0.956	1	0.970	0.985	0.970
**Specifity**	**Active**	0.990	0.984	1	0.972	0.984	1	0.975	0.984	0.975
	**Transitional**	0.997	0.969	1	0.994	0.946	0.994	0.989	0.938	0.989
	**Inactive**	0.975	0.991	1	0.967	1	1	0.975	0.991	0.975
**F1 score**	**Active**	0.967	0.959	1	0.956	0.952	1	0.956	0.915	0.956
	**Transitional**	0.983	0.948	1	0.941	0.931	0.996	0.949	0.916	0.949
	**Inactive**	0.988	0.977	1	0.970	0.967	1	0.972	0.985	0.972

**Table 15:** The models’ settings and performance metrics for classification.

№	Model	Trained Parameters	Number of Trainable Parameters	CT Dataset (%)	US Dataset (%)	MRI Dataset (%)	Dropout Rate	Learning Rate	Epochs	Batch Size	Regularization	Pre-Trained	Classification Layers
1	CNN	Entire Model	25,710,019	52.95	52.05	61.68	0.6, 0.4	0.00001	30	16	l2	imagenet	2FC+1Soft
2	VGG16	Entire Model	14,730,171	78.75	67.89	85.80	0.6, 0.4	0.00001	30	16	l2(0.01)	imagenet	2FCL+1Soft
3	VGG19	Entire Model	20,039,867	84.09	79.36	99.88	0.5, 0.3	0.00001	30	16	l2(0.01)	imagenet	2FCL+1Soft
4	Inception	Entire Model	21,829,915	97.36	93.64	100.0	0.6, 0.4	0.00001	30	16	l2	imagenet	
5	ResNet50	Entire Model	23,596,155	97.26	88.47	99.99	0.6, 0.4	0.00001	30	16	l2	imagenet	1FCL+Soft
6	MobileNet	Head only	38,523	79.67	60.35	90.86	0.5, 0.3	0.00001	30	16	l2	imagenet	
7	FNN	Customized	23,907,779	65.07	38.18	77.42	0.4	0.00005	30	32	l2	NA	
8	ViT	Entire Model	86,415,592	86.67	91.07	78.96	0.1	0.001	30	16	NA	INet-21k	1FCL
9	Inception+ResNet	Entire Model	46,203	96.78	94.65	99.99	0.6, 0.4	0.00001	30	16	l2(0.01)	imagenet	
10	CNN+ResNet	Entire Model	23,596,155	97.55	93.99	100.00	0.6, 0.4	0.00001	30	16	l2(0.01)	imagenet	

**Table 16:** Comparison of diagnostic tools for cyst classification: sensitivity and specificity scores.

Diagnostic Tool	Sensitivity (%)	Specificity (%)
Clinical Evaluation	NA	NA
US	0–98	95–100
CT Scan	90–100	∼100
MRI	90–100	∼100
ELISA (Serology)	80–94	85–95
Immunoblot (Serology)	88–96	97–99
Histopathology	100	100
PCR (Molecular Testing)	∼100	∼100
CNN+ResNet (CT)	96.0	98.1
CNN+ResNet (US)	95.4	97.8
CNN+ResNet (MRI)	100	100

## Data Availability

The research data underlying this manuscript have been sourced from previously published studies and datasets, all of which have been properly cited. The processed data can be obtained from the corresponding author upon reasonable request.

## Supplementary Materials

The following supporting information can be downloaded at: https://www.mdpi.com/article/10.3390/bioengineering12020181/s1, Supplementary Material 1: US dataset; Supplementary Material 2: MRI dataset.

## Abbreviations

The following abbreviations are used in this manuscript:

CE	Cystic echinococcosis
AE	Alveolar echinococcosis
WHO	The World Health Organization
WHO-IWGE	WHO Informal Working Groups on Echinococcosis
AI	Artificial Intelligence
ML	Machine learning
DL	Deep learning
ViT	Vision Transformer
ANN	Artificial Neural Network
CNN	Convolutional Neural Network
FNN	Feedforward Neural Networks
RNN	Recurrent Neural Network
SVM	Support Vector Machines
GAN	Generative Adversarial Network
KNN	K-nearest neighbors
CBCT	Cone Beam Computed Tomographic
US	Ultrasound
CT	Computed Tomography
MRI	Magnetic Resonance Imaging
TPR	True Positive Rate
HPC	High-Performance Computing
GPU	Graphics Processing Unit
SMOTE	Synthetic Minority Over-sampling Technique
