# Supplementary material for: A Study on Staging Cystic Echinococcosis Using Machine Learning Methods
Source: Bioengineering (Basel). 2025 Feb 13;12(2):181. doi: 10.3390/bioengineering12020181 (PMC11852189; doi:10.3390/bioengineering12020181)
Supplement: Supplementary file 1 [file bioengineering-12-00181-s001.zip › Dataset3_MRI.pdf]

Paper 1. [Yesilyurt, M., & Esdur, V. (2022). Anatomical-Based Imaging of Cystic Echinococcosis and Review of the Current Literature. The Eurasian Journal of Medicine, 54(1), S1.]

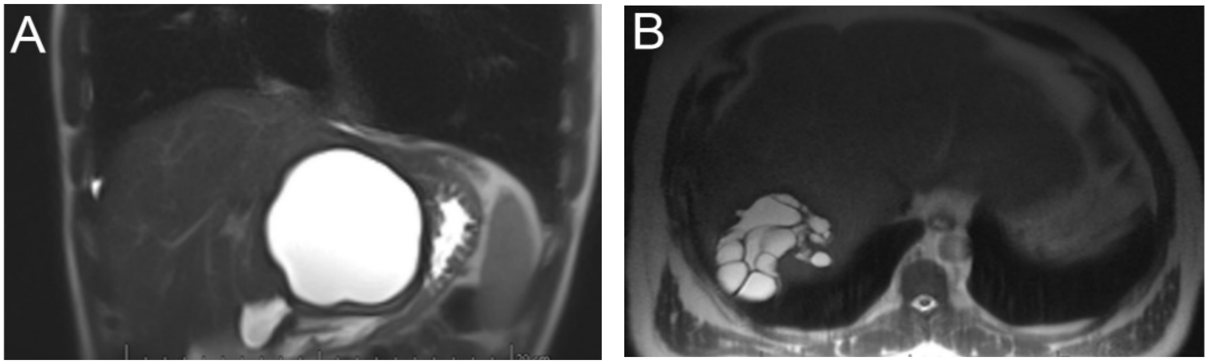

Figure 1.

figure 1	CE1	(A) Hyperintense hepatic cyst with double-line sign considered pathognomonic for World Health Organization (WHO) CE1 cysts, T2-weighted MRI.
	CE2	(B) Multivesicular cystic echinococcosis hepatic cyst WHO CE2 cyst, T2-weighted MRI.

Paper 2. [Stojkovic, M., Rosenberger, K., Kauczor, H. U., Junghanss, T., & Hosch, W. (2012). Diagnosing and staging of cystic echinococcosis: how do CT and MRI perform in comparison to ultrasound?. PLoS neglected tropical diseases, 6(10), e1880.]

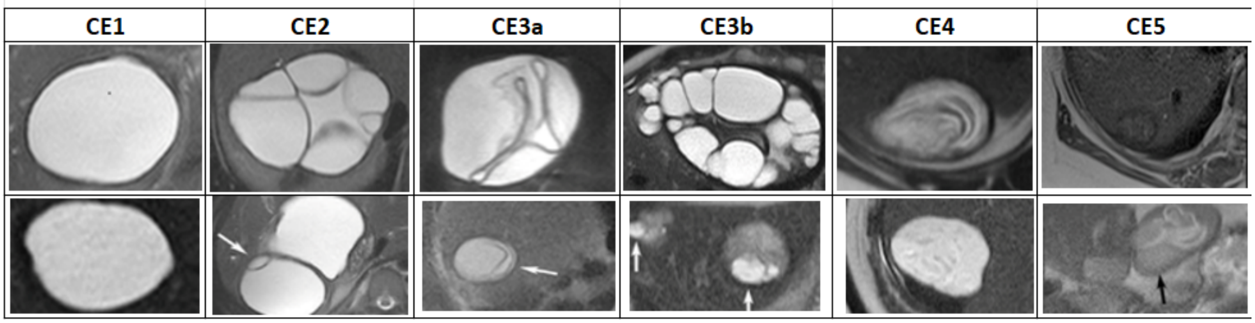

Figure 2.

Paper 3. Karakas, E., Uzunköy, A., Karakas, E. Y., Gundogan, M., Karakas, O., Boyaci, F. N., ... & Yucel, Y. (2014). Efficacy of diffusion-weighted magnetic resonance imaging in follow-up patients treated with open partial cystectomy of liver hydatid cysts. International Journal of Clinical and Experimental Medicine, 7(12), 5090.

CE1	Magnetic resonance images of 9-year-old girl patient with type 1 hydatid cysts.

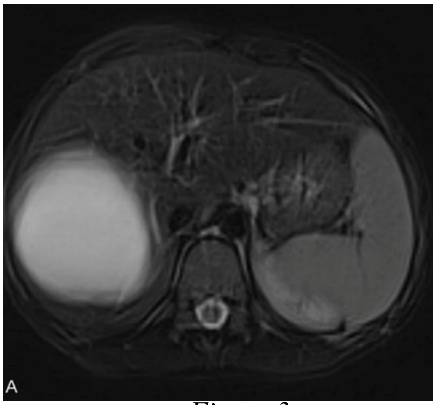

Figure 3.

Paper 4. Oruç, E., Yildirim, N., Topal, N. B., Kilicturgay, S., Akgöz, S., & Savci, G. (2010). The role of diffusion-weighted MRI in the classification of liver hydatid cysts and differentiation of simple cysts and abscesses from hydatid cysts. Diagnostic and interventional radiology, 16(4), 279.

Figure 4. CE4	A type 4 hydatid cyst. On axial T2-weighted HASTE MR image (a) the hyperintense mass that occupies the right lobe anterior segment and the left lobe medial segment of the liver is well depicted. This lesion has an irregular hypointense rim. Diffusion-weighted MR image (b) of the same lesion (b = 1000) exhibits a heterogeneous high signal intensity. Automated ADC map (c) shows an ADC value of $1.10x10^{-3}$. When the cut-off value is accepted as ≤ 1.76 for abscesses, this type 4 hydatid cyst was diagnosed as an abscess.

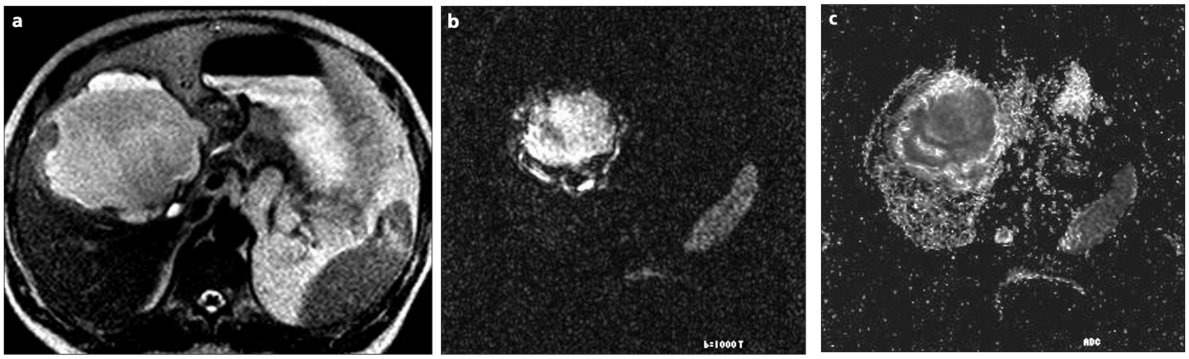

Figure 4.

Paper 5. Baykan, A. H., Aydin, E., Koc, M., Sahin, H., Karul, A., Baykan, M. E., ... & Erturk, S. M. (2024). Hydatid disease: imaging, treatment, and beyond. Clinical Radiology, 106748.

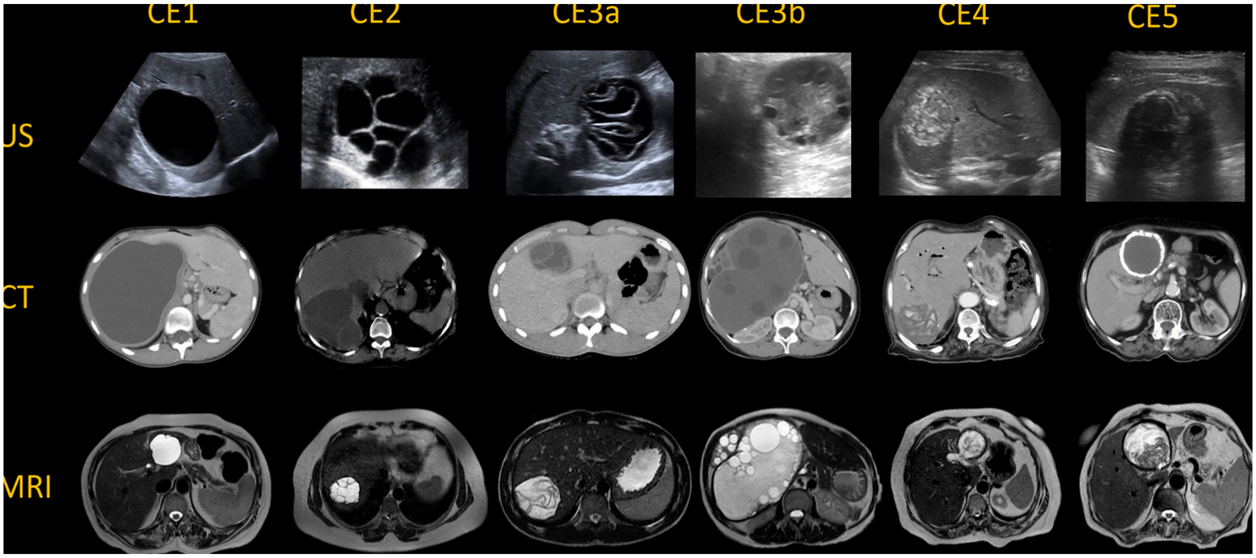

Figure 5.

Paper 6. Wen, H., Vuitton, L., Tuxun, T., Li, J., Vuitton, D. A., Zhang, W., & McManus, D. P. (2019). Echinococcosis: advances in the 21st century. Clinical microbiology reviews, 32(2), 10-1128.

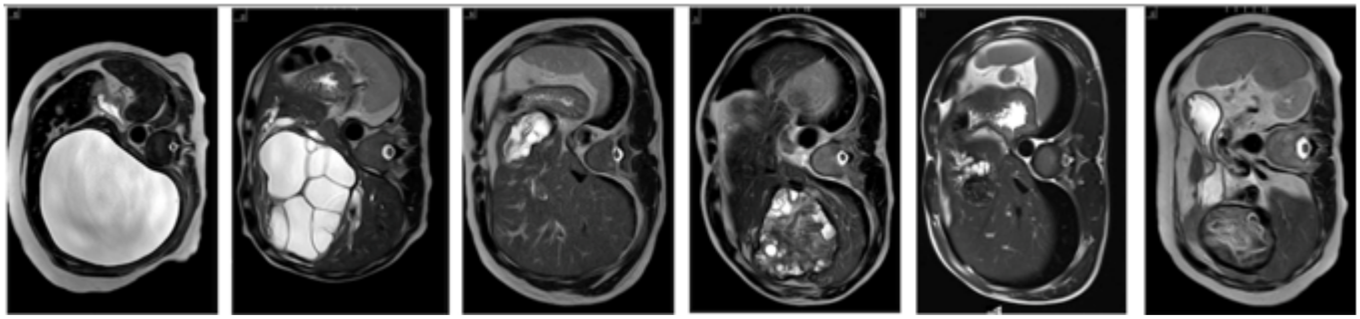

Figure 6.

Paper 7. Zhao, Z. M., Yin, Z. Z., Meng, Y., Jiang, N., Ma, Z. G., Pan, L. C., ... & Liu, R. (2020). Successful robotic radical resection of hepatic echinococcosis located in posterosuperior liver segments. World journal of gastroenterology, 26(21), 2831.

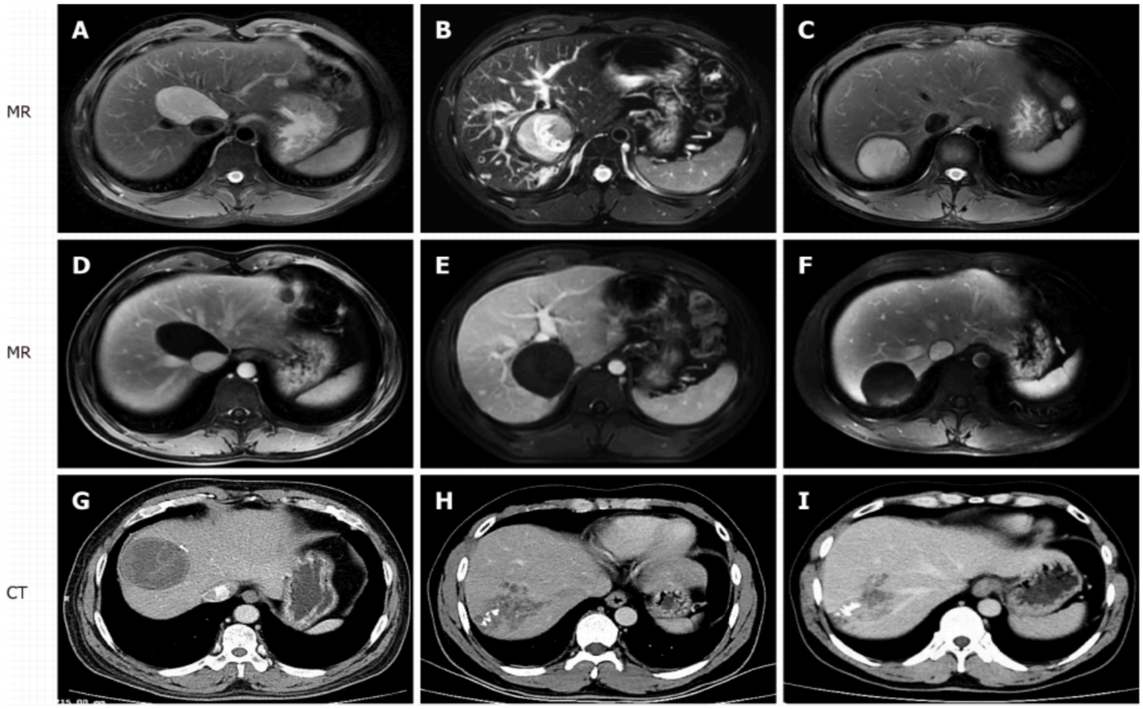

Figure 1 Contrast-enhanced magnetic resonance imaging and computed tomography manifestations of hepatic cystic and alveolar echinococcosis. A, D: Patient 1, cystic echinococcosis in caudate lobe; B, E: Patient 2, cystic echinococcosis in caudate lobe; C, F: Patient 3, cystic echinococcosis in segment VII; G: Patient 4, cystic echinococcosis in segment VIII; H, I: Patient 5, alveolar echinococcosis in segment VII/VIII. MR: Magnetic resonance; CT: Computed tomography.

Figure 7.

Paper 8. [Echinococcus granulosus Revisited: Radiologic Patterns Seen in Pediatric and Adult Patients]

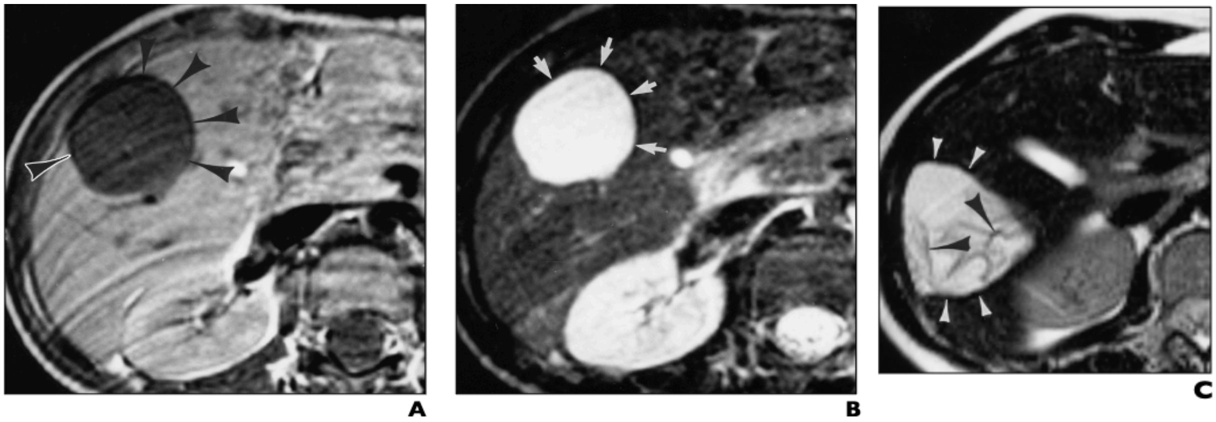

Figure 8.

Fig. 8. 8-year-old girl with Echinococcus granulosusinfection of liver.A, Axial intermediate-weighted spin-echo MR image (TR/TE, 2400/15) shows hydatid cyst in right liver lobe. Signalintensity is medium to low. Lesion is surrounded by low-intensity rim—typical rim sign (arrowheads) thought torepresent pericyst.B, Axial T2-weighted spin-echo MR image (2.4/90) showstypical appearance of hydatid cyst with high and homog-enous signal intensity. Lesion is surrounded by low-in-tensity rim (arrows).CBA

Paper 9. Haouimi A Hepatic hydatid cyst - stage CE 4. Case study, Radiopaedia.org (Accessed on 04 Jan 2025) https://doi.org/10.53347/rID-98738

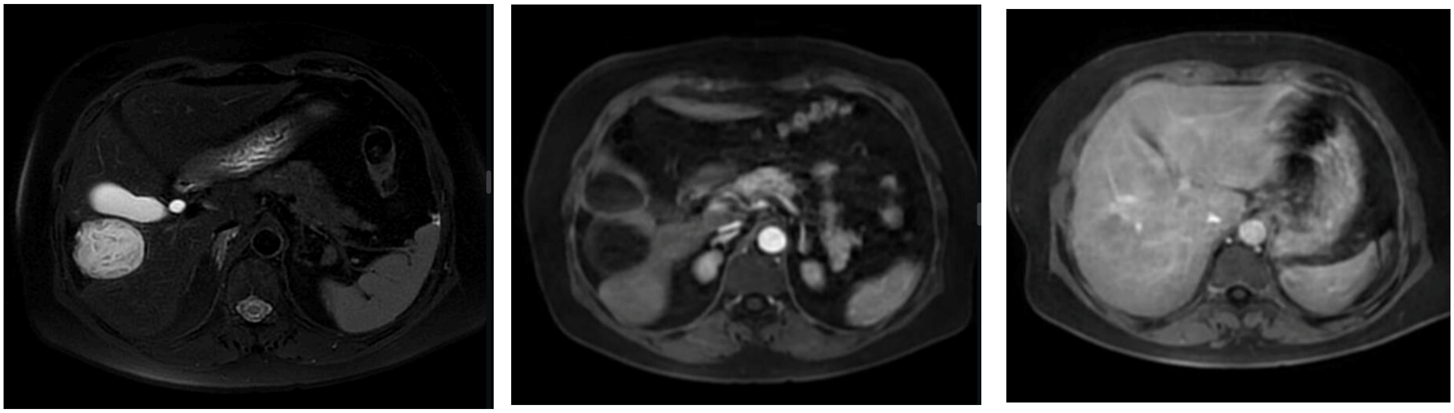

Figure 9.

MRI and ultrasound features of a hepatic hydatid cyst stage CE 4, according to the 2001 WHO classification of hepatic hydatid cysts.

		Paper1 []	Paper2 []	Paper3 []	Paper4 []	Paper5 []	Paper6 []	Paper7 []	Paper8	Paper9	Total
Active	CE1	1	2	1		1	1		3		14
	CE2	1	2			1	1				
Transitional	CE3a		2			1	1	6			14
	CE3b		2			1	1				
Inactive	CE4		2		3	1	1			3	14
	CE5		2			1	1				
		2	12	1	3	6	6	6	3	3	42
